# Cognitive state monitoring for neuroadaptive information visualization

**DOI:** 10.3389/fnhum.2026.1793651

**Published:** 2026-05-21

**Authors:** Sebastian Idesis, Michalis Kassinopoulos, Miguel Barreda-Ángeles, Luis A. Leiva, Ioannis Arapakis

**Affiliations:** 1 Telefónica Innovación Digital, Barcelona, Spain; 2 Barcelonaβeta Brain Research Center, Barcelona, Spain; 3 Joint Research Centre (JRC) of the European Commission, Ispra, Italy; 4 University of Luxembourg, Esch-sur-Alzette, Luxembourg

**Keywords:** neuroadaptive systems, mental fatigue, mental workload, stress, mind wandering

## Abstract

In today's digital world, we face an overwhelming influx of information daily. Information Visualisation (InfoVis) serves as a promising approach to alleviate this “information overload” issue by providing timely, effective, and adaptive visual representations. Despite its potential, previous research has under-explored the critical role of users' cognitive states in facilitating more natural interactions. This article bridges this research gap by first presenting a comprehensive review of current methodologies for assessing cognitive states through neurophysiological signal analysis. Specifically, we focus on InfoVis systems and the effects of certain cognitive states (mental fatigue, mental workload, stress, and mind wandering) on user performance. We also review strategies for assessing these states using non-invasive brain imaging modalities (EEG, fNIRS) and peripheral physiological measurements (HRV, EDA, and eye activity). Our critical analysis identifies the most effective tools for detecting real-time changes in cognitive processes, setting the groundwork for neuroadaptive InfoVis systems that promise enhanced user engagement and decision-making efficacy. Ultimately, this work advances our understanding of perception, provides best practices for multisensory integration in computing systems, and can inspire future technological innovations.

## 1 Introduction

The modern digital world inundates users with an overwhelming array of choices, the sheer number of which presents a well-known problem to our perception system: *information overload* (Iyengar and Lepper, 2006; Diehl and Poynor, 2008; Danziger et al., 2011; Chernev et al., 2015). We know from experience that we are surrounded by more information than we can handle and comprehend at any given time. The human nervous system, therefore, has to make decisions about what to process and what to filter, and in what order they should be prioritized (Eysenck and Keane, 2015). To top that, our perceptual system contains limited-capacity stages at which it can process only a certain amount of information at any given time, resulting in processing bottlenecks. Sequential choices, and the apparent mental depletion they evoke, also increase people's tendency to resort to sub-optimal policies (Levav et al., 2010). In addition, the consideration of the costs and benefits (Padoa-Schioppa, 2011) of each option only exacerbates this modern-day challenge.

Information Visualisation (InfoVis) technologies provide a way to expand and enhance our innate cognitive abilities, and enable us to accomplish a range of tasks, from simple (e.g., company revenue assessment) to intractable (e.g., worldwide air-traffic control) ones. In this review, InfoVis refers to digital, computer-mediated visual representations of data/information designed to support understanding, exploration, and decision-making. We therefore exclude arbitrary non-digital arrangements. Although some interfaces may include multimodal cues (e.g., auditory or tactile), our focus is on visual information presentation and its cognitive-state-driven adaptation. For example, interactive and responsive visualizations now allow users to dynamically manipulate and explore data, while recent developments in augmented and virtual reality technologies facilitate the exploration of 3D visualizations in a more intuitive and immersive manner. However, depending on the timing and context, we may have a greater or lesser ability to make rapid and effective decisions, yet our ability to do so based on fast-flowing streams of data may be decisive. From emergency rooms and autonomous cars to operational command centers, a clear understanding, rapid assessment, and decisive actions based on the available information can make a true difference.

**Figure 1:**
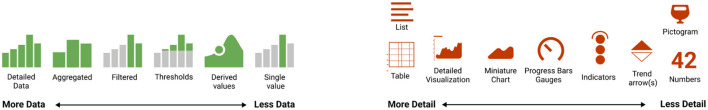
Visualization patterns help calibrating information exposure. Source: https://dashboarddesignpatterns.github.io/.

While using visualizations to mitigate this effect has been widely discussed as a possible countermeasure (Perkhofer et al., 2016), the potential of InfoVis systems has not yet unlocked their true potential to best assist the user in critical decision-making tasks. With the volume, variability, and velocity of today's data streams, our ability to successfully respond to critical events in InfoVis depends on the dashboard controls we have available, and how quickly we can draw actionable insights from them (Figure 1). For example, dashboard controls provide visual representations of non-physical and abstract concepts, such as statistical trends. However, for the non-expert human operator, it is unnatural to think in purely numerical and mathematical abstractions. Our brains are wired to recognize patterns and, as good as humans may be at this form of perception and cognition, some of the most important visual patterns may not be directly available for computation.

We argue that Artificial Intelligence (AI) enhanced with context-aware capabilities will play a key role in advancing next-generation InfoVis systems and addressing the aforementioned challenges. Although affective sensing may be relevant in some adaptive interfaces, this review focuses primarily on cognitive-state monitoring. Achieving this requires models capable of learning robust signatures of mental processes across users and contexts. Recent advances in Deep Learning (DL) contribute to this goal, and their use will be discussed within each sensing modality throughout this article. Neuroscience and neurotechnology have been among the earliest and most influential domains in the adoption and development of brain-signal sensing and modeling approaches, with increasing translation to consumer products. Commercial development and exploitation of Brain Computer Interface (BCI) implants for communication is increasing rapidly (Won et al., 2021), not only at the hardware (e.g., the well-funded companies Neuralink, Synchron, Paradromics, and Cortec Neuro) but also at the sensor level.

These developments are relevant across healthcare and rehabilitation, human-computer interaction, neuroergonomics/ workplace monitoring, and consumer wellness. Progress depends not only on sensing hardware, but also on data and analytics pipelines for signal acquisition, processing, interpretation, and governance. In this review, we focus specifically on their relevance for cognitive-state-aware adaptive InfoVis systems.

Developing such systems has been an out-of-reach vision, only realized in science fiction novels and movies alike. Dehaene et al. (2017) argued that most AIs fall within a C0 stage of “unconscious processing,” although we can find examples of so-called super-human behavior in computer vision (Esteva et al., 2017), machine translation (Hassan et al., 2018), and game playing (Silver et al., 2018), among other tasks. However, truly intelligent systems must be equipped with a new form of consciousness. According to Dehaene's principles, reaching a C1 stage of global availability and potentially a C2 stage of self-monitoring or meta-cognition is essential. These capabilities will allow machines to enhance their potential as truly intelligent partners.

Cognitive and affective state monitoring for guiding adaptations in InfoVis systems has been used in several applications like augmenting human engagement in games and assisting learning process in educational software (Aranha et al., 2021). However, limited work (Maat and Pantic, 2006; Hirshfield et al., 2009) has been done examining the benefits of this approach in critical-safety domains, such as air traffic control, driving and military operations, where failure of the user interaction with the InfoVis system has severe consequences. This is somewhat surprising given the evidence that cognitive processes are affected by the format of visual interface and such effects can be detected with neurophysiological signals (Hirshfield et al., 2011; Peck et al., 2013; Lukanov et al., 2016). To assist human activity using intelligent visual adaptations, two challenges must be addressed: (i) assessing the user's cognitive state, and (ii) defining how this information should guide visual adaptations. In this article, we address these issues by reviewing key cognitive states and the neurophysiological signals used to monitor them, while also highlighting how each state influences interaction and decision-making within InfoVis environments.

In this article, we address the challenge of cognitive state monitoring by providing an updated review of key cognitive states and neurophysiological signals from the last two decades (2005–2025). By tackling this challenge, we enable future research to leverage the strengths of our visual functions while mitigating the limitations of our cognitive functions. Additionally, by designing adaptive InfoVis systems supported by AI with social and collaborative features, we enhance the orchestration of complex collaborative work across various scales.

## 2 Related work

Although the potential of cognitive state monitoring in the design of InfoVis systems has yet to be fully recognized, its advantages in various other applications have long been established. For example, cognitive state monitoring has been shown to enhance user engagement in video games (Bontchev, 2016) and museum tours (Abdelrahman et al., 2015), facilitate learning in educational environments (Gerjets et al., 2014; Yuksel et al., 2016), and improve the behavior of robotic agents (Kim et al., 2017; Salazar-Gomez et al., 2017). Furthermore, insights into cognitive state have been demonstrated to boost performance in safety-critical systems (Liu et al., 2013) and enhance the effectiveness of rehabilitation (Andrade et al., 2016).

Physiological computing has enormous potential to innovate Human–Computer Interaction (HCI) by extending the communication bandwidth to enable the development of smart technology (Fairclough, 2009) and progress toward mixed-initiative architectures, as well as more efficient human-robot interaction systems (Roy et al., 2020). In the context of InfoVis, these same physiological signals offer a way to adapt visual representations to the user's cognitive demands, strengthening the link between sensing and adaptive visual design. Concretely, neuroadaptive systems are designed to make adaptations to the user interface based on closed-loop implicit monitoring and analyses of neurophysiological data (Fairclough, 2022). Lee et al. (2024) argued that, currently, data is only transmitted from human to machine, thus an important research gap remains to enable mutual understanding between humans and machines. In this context, adaptive interfaces can play a crucial role in developing new interaction paradigms, especially when improving performance or user experience (Chiossi et al., 2022). For example, mental state-based adaptive interface design can incorporate particular characteristics of the domain of application (Salomone et al., 2023; Hinss et al., 2022). Muñoz et al. (2021) proposed a taxonomy for intelligent adaptation via physiological metrics that groups several adaptive systems. In what follows, we discuss relevant work that, to our knowledge, has set important precedents in the InfoVis community.

Lallé et al. (2015) predicted the user's learning curve when working with interactive InfoVis for decision making. They found that various eye-tracking features such as fixation rate or pupil dilation can help model a user's initial expertise with a visualization, as well as their expected learning rate for the related skills. These insights could inform adaptive visualizations for supporting the so-called novice-to-expert transition in completing visual tasks (Steichen et al., 2020).

Traditionally, InfoVis systems have followed a one-size-fits-all model, without accounting for individual user preferences, abilities, or contexts (Steichen and Fu, 2019). However, research has shown ample evidence that individual characteristics can have significant effects on a user's processing of visual information (Mawad et al., 2015; Raptis et al., 2016). For example, Steichen and Fu (2019) explored the effect of an individual user's cognitive style on InfoVis performance. They found that the user's cognitive style and experience plays a significant role when interacting with InfoVis systems, and that different visualization aids are chosen to different degrees. Previous work has also investigated the effect of human factors, such as cognitive processing capabilities (Germanakos et al., 2009). There are also many examples showing that there is an effect of personality, cognitive abilities, and expertise on a user's performance with different visualizations (Velez et al., 2005; Green and Fisher, 2010; Toker et al., 2012; Luo, 2019).

For years, the most common adaptation technique in InfoVis has been to recommend alternative visualizations (Grawemeyer, 2006; Gotz and Wen, 2009). Recently, Kong and Agrawala (2012) developed a system that could dynamically add overlays to a visualization in order to aid chart understanding. Similarly, Carenini et al. (2014) focused on a subset of “visual prompts” that could be used for highlighting specific data points that are relevant to the user's current task. Karolus et al. (2017) explored the potential of using a user's gaze properties to detect whether the information is presented in a language the user understands. They found that a few seconds of recorded gaze data is sufficient to determine if a user can speak the displayed language. Spiller et al. (2021) investigated the predictive power of eye gaze data in visual search tasks, focusing on the initial ten seconds of user interaction as a foundation for developing user-adaptive InfoVis systems. Their findings reveal that the majority of users acquire the critical information necessary for successful task completion within the first second of engagement, underscoring the relevance of gaze-based signals for designing adaptive visual interfaces that respond to users' cognitive state in real time. Scanpath analysis has also been explored directly within InfoVis contexts, where gaze sequences help characterize how users navigate graph-based visualizations (Lopez-Cardona et al., 2025a).

Müller et al. (2022) studied the noticeability of desktop notifications during realistic interaction tasks. They found that the visual importance of the background at the notification location significantly impacts whether users detect notifications. Moreover, they introduced “noticeability maps” to help designers better balance notification design and saliency. Finally, recent work by Chiossi et al. (2022) emphasizes the potential of multimodal adaptive systems, when integrated with sensor fusion technology, to comprehensively capture user context in future applications. This finding marks the importance of revisiting the state-of-the-art in computing systems that can capture the cognitive processes involved in decision-making tasks, when interacting with InfoVis systems. In this article, we review existing literature on the topic, highlighting current limitations and proposing solutions to overcome these challenges by integrating bio-physical measurements with modern analytical techniques, including the role of DL within each sensing modality.

In sum, neurophysiological and peripheral signals offer several advantages over traditional approaches, such as standardized questionnaires, since the signals provide continuous data streams and their recordings do not disrupt the state being measured. However, the effectiveness of this method depends on the ecological validity of the recordings and their interpretations (Krol and Zander, 2017). Given that neurophysiological sensing technology is becoming more and more compact and affordable, the feasibility of integrating such sensors in everyday applications, such as in-car driver fatigue detection, is increasingly within reach.

## 3 Neurophysiological and peripheral measurements

**Figure 2:**
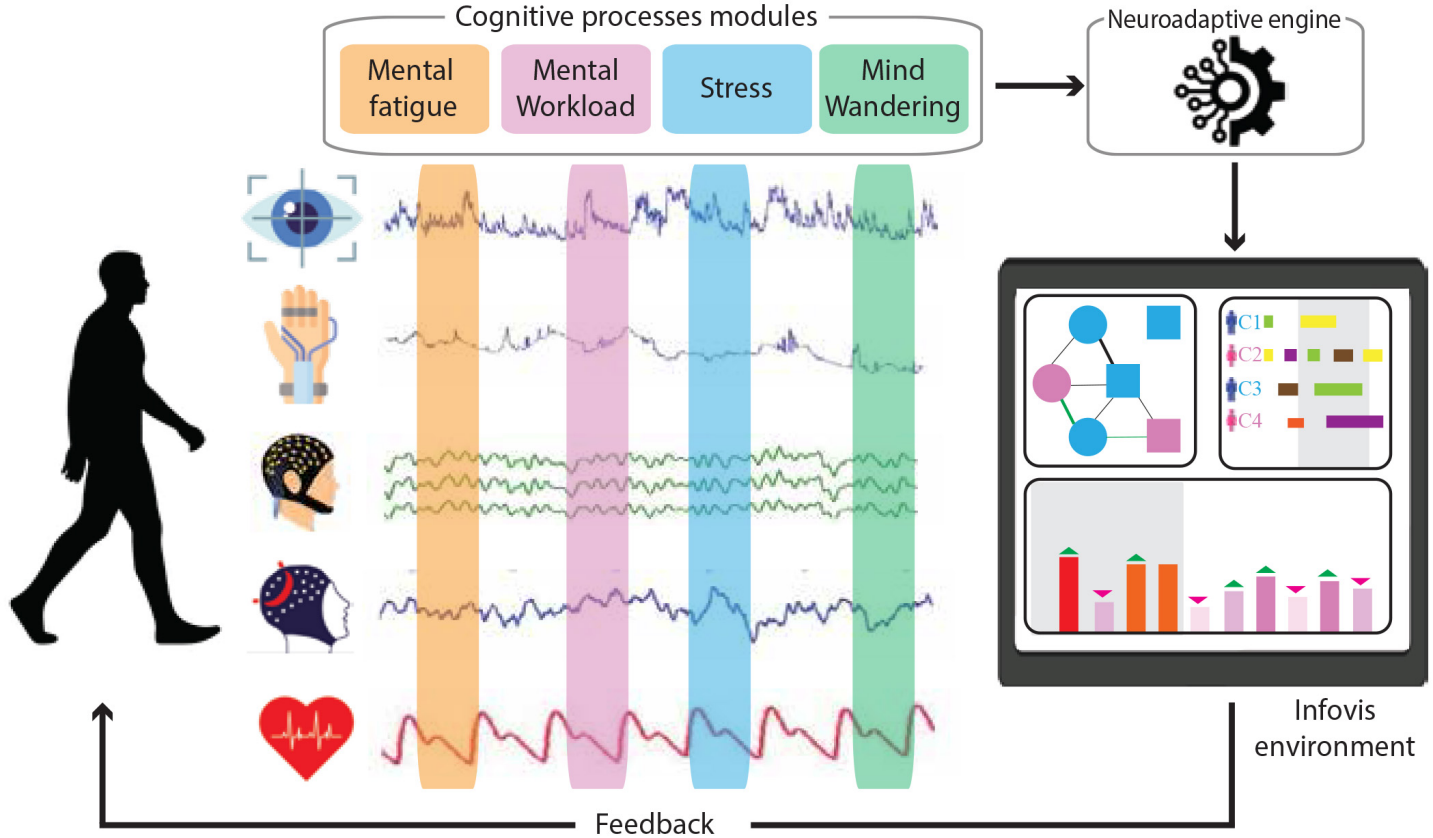
Conceptual overview of a neuroadaptive system for monitoring and responding to cognitive states. Multimodal physiological and behavioral signals are continuously acquired during user activity and processed to infer cognitive process, including mental fatigue, mental workload, stress, and mind wandering. These inferred states drive adaptive feedback and real-time adjustments within a neuroadaptive InfoVis environment, forming a closed-loop human–system interaction.

The importance of employing neurophysiological and peripheral measurements in cognitive state analysis is well-documented in current research (reported in this section) due to the complementary strengths and capabilities of these techniques. Combining these methods offers a comprehensive toolset for studying cognitive states by leveraging the strengths of each while mitigating their individual limitations. This multimodal approach enhances data accuracy and richness, enabling deeper insights into the complex interactions between brain activity, physiological responses, and behavior. In the context of InfoVis, these measurements are particularly relevant because they allow real-time monitoring of how users process and interact with visual information (Figure 2).

We note that we only considered for analysis affordable and wearable technology that emphasizes usability and real-world adaptability for InfoVis. As such, other sensing approaches were not included in the current article due to their cost and portability, such as functional Magnetic Resonance Imaging (fMRI), Magnetoencephalography (MEG), or Positron Emission Tomography (PET). Furthermore, neuromodulation and brain stimulation approaches (e.g., TMS, tDCS, and related techniques) are outside the scope of this review, which focuses on sensing and monitoring modalities for cognitive-state-aware adaptive InfoVis.

### 3.1 EEG

Electroencephalography (EEG) has been one of the most powerful techniques for studying brain activity in a non-invasive manner. It measures the electrical activity generated by large populations of neurons in the cortex, recorded non-invasively through electrodes placed on the scalp. These voltage fluctuations reflect synchronous neural activity and can be tracked with high temporal precision (Baillet, 2017).

EEG recordings can be used in real time to infer cognitive state [e.g., mental workload (Aric et al., 2016)] and support motor-imagery-based BCI control (Bi et al., 2013). Owing to its temporal precision, robustness, and portability, EEG remains a preferred modality for BCIs compared with less practical techniques such as fMRI or MEG (Sanei and Chambers, 2013). As a consequence, EEG is also widely used in clinical and diagnostic contexts.

BCI has been used extensively for enabling individuals with severe motor and communicative disabilities, like locked-in syndrome (LIS) patients, to control external robotic devices (e.g., a prosthetic forearm) or communicate through a computer (e.g., through handwriting imagination (Willett et al., 2021). However, in the last decade there has been an increasing interest in using EEG-based BCI for non-medical purposes, with applications ranging from measuring drowsiness levels during driving (Craig et al., 2012) to improving adaptive automation for air traffic control, based on mental workload measurements (Aric et al., 2016). These properties make EEG a valuable modality for InfoVis systems, where detecting ongoing fluctuations in users' cognitive state can support real-time visual adaptations.

EEG acquisition is typically standardized using established electrode-placement systems (e.g., 10–20/10–10), and channel counts vary by device and application. Because this review focuses on neuroadaptive InfoVis rather than introductory EEG methodology, we only summarize signal representations most relevant to adaptive systems.

The most widely used strategy in the literature for interpreting EEG data has been the spectrum band approach which, interestingly, is also the first one utilized in the early EEG studies. Specific EEG frequency bands have been associated to sleep stages and levels of alertness, and are commonly defined as follows (Platt and Riedel, 2011; Martinek et al., 2021):

**Delta (1–4 Hz):** prominent during deep sleep.

**Theta (4–8 Hz):** increases during drowsiness and early sleep stages.

**Alpha (8–12 Hz):** strongest during relaxed wakefulness, especially with eyes closed.

**Beta (12–30 Hz):** associated with alertness and active mental processing.

**Gamma (30–80 Hz):** linked to high-level cognitive tasks such as working memory and reading.

A sliding-window approach is employed when computing the Power Spectral Density (PSD) in EEG to track variations in the power of each frequency band. Typically a 30 sec time window is considered, although shorter time windows (e.g., 3 sec) are also sometimes preferred for achieving higher temporal resolution (Olbrich et al., 2009).

In practice, EEG-based adaptation may use other approaches besides frequency-domain features, like time-locked responses (such as ERPs), and paradigm-specific signatures (such as SSVEPs), depending on the interaction design and inference target. Feature and processing choices should therefore be driven by the target cognitive process, temporal constraints, and robustness requirements. Recent work has also explored using DL models to learn informative EEG patterns directly from raw signals, reducing the need for handcrafted features.

### 3.2 fNIRS

Functional Near-InfraRed Spectroscopy (fNIRS) is a non-invasive optical imaging technique used across a broad range of populations in both research and clinical contexts, including healthy participants as well as individuals with neurological, neurovascular, or other medical conditions, depending on the application and study design (Boas et al., 2014). It offers advantages over MRI and fMRI in terms of cost-effectiveness and portability. fNIRS utilizes Near-Infrared (NIR) light's ability to penetrate the human scalp and skull, reaching the underlying cortical neuronal tissues. Detectors capture backscattered light, enabling measurement of changes in oxygenated (HbO2) and deoxygenated (HbR) hemoglobin concentrations associated with neuronal activation. This technique provides valuable insights into cortical hemodynamics and, by extension, neural activity. Its functional interpretation relies on neurovascular coupling, i.e., the relationship between local neural activity and subsequent changes in cerebral blood oxygenation and blood volume. These characteristics make fNIRS suitable for studying how users process visual information, offering a complementary tool for understanding cognitive states during InfoVis interaction. Representative fNIRS features include changes in oxyhemoglobin (HbO) and deoxyhemoglobin (HbR), total hemoglobin (HbT; often used as a proxy for blood-volume-related changes), and temporal hemodynamic features such as response amplitude, slope, latency-to-peak, and recovery dynamics.

The instrumentation of fNIRS systems has matured significantly since the first functional studies, shifting from single- to multi-channel systems (Boas et al., 2014). In addition, the fNIRS community has rapidly grown in the past few decades establishing the important role of fNIRS for studying the function of the healthy and pathological brain (Boas et al., 2014; Pinti et al., 2020). fNIRS systems are less prone to motion artifacts as compared to other neuroimaging modalities, while some systems also offer portability due to their small size, which allows them to be wearable and wireless (Perrey, 2008; Boas et al., 2014). As such, fNIRS researchers have recently started exploring the potential of this technology for studying brain function in more naturalistic settings than the lab, and without restricting the participants from moving or walking as they would normally do in real-life scenarios (Pinti et al., 2018). Moreover, fNIRS has been used in neuroergonomics for assessing the cognitive state of individuals at naturalistic work settings although this area of research is still in its infancy (Lohani et al., 2019). Neuroergonomics refers to the study of brain and behavior in relation to human performance in real-world tasks and environments, integrating neuroscience, human factors, and cognitive engineering (Parasuraman, 2003).

Interpreting fNIRS measurements relies on how NIR light is absorbed and scattered by brain tissue. fNIRS systems operate in the optical window (around 650–1,350 nm), where tissue absorption is low enough to allow light to reach the cortex. By measuring changes in how much light is returned to the detectors, fNIRS provides estimates of fluctuations in HbO2 and HbR (Scholkmann et al., 2014). Therefore, by quantifying changes in concentrations of HbO2 and HbR, fNIRS allows us to infer changes in neuronal activity that precede the haemodynamic changes (Scholkmann et al., 2014). When a brain area shows increased neuronal activity, due to for example some cognitive task, the demands of that area in nutrients such as oxygen and glucose are increased. These increased demands lead to an increase in cerebral metabolic rate of oxygen. In addition, the release of carbon dioxide is increased as carbon dioxide is a potent vasodilator that can mediate the increases in cerebral blood flow and, hence, speed up the transport of fresh blood at the capillary beds. Although still not fully understood, increases in blood flow are much higher than what is required for the demands in oxygen (Iadecola and Nedergaard, 2007). As a result, neuronal events are followed by elevations in blood flow leading to increases in levels of HbO2 and cerebral blood volume (CBV) and decreases in levels of HbR. Fluctuations in the aforementioned haemodynamic parameters are more prominent in the small blood vessels (arterioles, capillaries and venules) and can be detected with an fNIRS system. However, the NIR light that travels through the brain tissues, apart from being absorbed from substances present on its path, it also gets strongly scattered leading to further light attenuation (Scholkmann et al., 2014). In continuous-wave (CW) fNIRS, signals are typically used to estimate relative changes in oxyhemoglobin and deoxyhemoglobin concentrations over time. In contrast, frequency-domain (FD) and time-domain (TD) fNIRS can support estimation of absolute optical properties and, under appropriate models, absolute hemoglobin concentrations. Accordingly, real-world and wearable fNIRS applications are not limited to CW systems, and newer wearable platforms also include TD-fNIRS implementations. Furthermore, in order to analyze the obtained information, recent fNIRS studies have also incorporated DL models to learn discriminative patterns directly from haemodynamic signals, complementing traditional feature-based approaches (Tanveer et al., 2019a; Karmakar et al., 2023; Eastmond et al., 2022).

The changes of HbO2 and HbR in response to a brief stimulus have a characteristic shape termed Haemodynamic Response Function (HRF). Typically, for a brief stimulus, the HRF initially increases reaching its peak at about 5–6 sec after the stimulus onset and then returns to its baseline with a delay of about 16 sec (Pinti et al., 2020). However, small variations in the shape have been reported across different brain regions, experimental stimuli and ages. With respect to the instrumentation, a light detector is placed at a certain distance from the NIR light source to collect the backscattered light and measure changes in light attenuation. The measured light can be used to infer brain activity from a “banana-shaped” brain volume along the path between the source and the detector (Quaresima and Ferrari, 2019). A source-detector pair is considered to be most sensitive to haemodynamic changes located at the mid-point between the source and the detector, at a depth of around the half of the source-detector distance (Patil et al., 2011). The penetration depth of the light increases as the source-detector increases (Pinti et al., 2020). However, the longer is the source-detector distance the longer is also the path that the light needs to travel. Thus, longer source-detector distances lead to stronger light attenuation due to absorption and scattering, and eventually to lower Signal-to-Noise Ratio (SNR). Typically, fNIRS studies consider a 30–35 mm distance for adults and a 20–25 mm distance for infants to ensure a good trade-off between penetration depth and SNR. Considering these distances, brain activity can be detected from the cerebral cortex with a depth sensitivity of about 1.5 cm and a spatial sensitivity up to 1 cm (Quaresima and Ferrari, 2019).

### 3.3 HRV

Heart rate varies on a moment-to-moment basis in response to ongoing changes in physical and cognitive demands, and is regulated by the autonomic nervous system, specifically the sympathetic and parasympathetic components (Rajendra Acharya et al., 2006). An increased activity of the sympathetic component is typically characterized by elevated Heart Rate (HR) and decreased Heart Rate Variability (HRV), while increased parasympathetic activity is characterized by decreased HR and increased HRV. Existing evidence demonstrates that the evaluation of the momentary changes in HRV can provide surrogates of fluctuations in cognitive processes, and particularly in stress and attention levels (Alaimo et al., 2020; Qin et al., 2021). In the context of InfoVis, HRV can provide continuous, unobtrusive indicators of how users respond to visual complexity and cognitive demand during interaction.

Experiments in laboratory settings often employ electrocardiography (ECG) for monitoring cardiac activity which involves the placement of three or more lead cables on the upper chest and lower abdomen. ECG measures the electrical activity that arises from heart muscles during cardiac contractions and passes through the soft tissues to the superficial skin. The basic pattern of the ECG consists of a series of waves (deflections of electrical activity), including the R wave that reflects depolarisation of the main mass of the ventricles and, thus, it is the largest wave. As such, the HR is defined based on the R-to-R (RR) intervals across the cardiac cycles. The HR is also often measured in a laboratory with photoplethysmography (PPG) sensors, commonly placed on the ear, wrist, or finger, depending on the device and application. PPG is an optical-based device that detects changes in blood volume and is used for determining the HR based on the peak-to-peak (PP) intervals of the acquired signal. Although PPG measures a haemodynamic signal rather than electrical activity of the heart, it provides similar HRV traces with the ones obtained using ECG (Selvaraj et al., 2008). In addition, cardiac activity is sometimes monitored with wearable devices (e.g., smartwatches) that are well tolerated by participants and are more suitable for naturalistic experiments that involve movement (Nelson and Allen, 2019).

Several HRV measures have been proposed in the literature which can be broadly categorized into time-domain and frequency-domain measures (Shaffer and Ginsberg, 2017). For time-domain measures, the fluctuations in instantaneous HR or the time intervals between adjacent heartbeats are first determined. In some cases, ectopic beats arising from arrhythmic events are detected and discarded, keeping only the normal heartbeat intervals that result from sinus node depolarizations. Then, a statistical variable is calculated based on these time-series. For example, the standard deviation of normal heartbeat intervals is estimated for deriving the SDNN measure (see list below). In the case of frequency-domain HRV measures, the time-series of beat-to-beat intervals is first derived and resampled to a timeline with equidistant intervals (e.g., 100 ms). Subsequently, the PSD is estimated using non-parametric (e.g., fast Fourier transform) or parametric techniques (e.g., Welch's method) that allows the separation of HRV into distinct frequency bands, namely the ultra-low (ULF; < 0.003 Hz), very-low (VLF; 0.0033–0.04 Hz), low (LF; 0.04–0.15 Hz) and high (HF; 0.15–0.40 Hz) frequency bands (Shaffer and Ginsberg, 2017). Below we summarized the time-domain and frequency-domain HRV measures most commonly used in neuroergonomics for assessing cognitive states:

**Mean RR interval (ms):** This measure corresponds to the average beat-to-beat interval of the cardiac recording within a predefined period. A longer RR interval reflects higher parasympathetic activity.

**Mean heart rate (bpm):** This is the mean number of heart beats per minute during a predefined period. Higher heart rate indicates increased sympathetic activation.

**RMSSD (ms):** The root mean square of successive differences between normal heartbeat intervals. Higher RMSSD values reflect stronger parasympathetic activity.

**SDNN (ms):** The standard deviation of normal heartbeat intervals. In short-term recordings (approximately 5 min), SDNN is mainly driven by parasympathetic activity.

**HF power (ms**^2^**):** High-frequency power (0.15–0.40 Hz). This band reflects parasympathetic activity and is often called the “respiratory band,” as it is strongly influenced by respiration-related heart rate variations.

**LF power (ms**^2^**):** Low-frequency power (0.04–0.15 Hz). Under resting conditions, LF power primarily reflects sympathetic (baroreceptor) activity, although parasympathetic contributions can occur at slow breathing rates.

**LF/HF ratio:** The ratio between the low-frequency and high-frequency components. A high LF/HF ratio indicates sympathetic dominance, whereas a low ratio suggests parasympathetic dominance.

### 3.4 EDA

Electrodermal Activity (EDA) refers to changes in skin conductivity due to sympathetic nervous system activity. The activation of the sympathetic branch of the nervous system stimulates the production of sweat in the eccrine glands located in the palms of the hands and soles of the feet, increasing skin conductivity in these areas. Thus, by passing a small electric current between two electrodes placed in these areas (usually on the palmar side of two fingers) it is possible to measure such changes in conductivity, obtaining a proxy of sympathetic activation.

The result is a non-stationary signal, in which two components can usually be distinguished: a tonic component—often referred to as Skin Conductance Level (SCL)—which represents the overall level of skin conductance and varies relatively slowly, and a phasic component—Skin Conductance Responses (SCR)—which involves momentary, relatively faster, increases over the tidal drift of the SCL (Dawson et al., 2017).

Commonly used EDA measures focus on both tonic and phasic activity. Typical phasic features include the number of SCRs, their amplitude, rise time, and recovery time, as well as Area Under the Curve (AUC) of the response. In addition, several studies have examined the complexity of the EDA signal, which can help discriminate between different arousal states or stress conditions (Nardelli et al., 2022).

In summary, representative EDA features include skin conductance level (SCL; tonic component), skin conductance responses (SCRs; phasic component), SCR amplitude, SCR count/frequency (including non-specific SCRs, depending on the paradigm), timing features (e.g., latency, rise time, recovery time), and tonic/phasic decomposition-derived features when signal separation is applied before feature extraction.

The study of EDA in response to external stimulation dates back to the 19th century and, since then, this has been one of the most popular psychophysiological signals (Dawson et al., 2017). This is due, in part, to the relative simplicity and the low cost of the equipment needed to collect it but also to the fact that EDA can provide information about numerous mental constructs involving changes in sympathetic activity.

As a proxy of sympathetic activity, EDA is considered “a pure arousal indicator” (Nardelli et al., 2022), which, in turn, underlies various cognitive and emotional processes (Westbrook and Braver, 2015; Lang and Bradley, 2010). In InfoVis contexts, EDA can provide continuous indicators of users' arousal and engagement during interaction with visual displays.

### 3.5 Eye activity

Eye movements are one of the most powerful aspects of interaction. Tracking eye movements can be used instead of traditional inputs like a mouse, keyboard, or touch-pads, contributing to interesting HCI applications. This technology monitors eye movements, allowing researchers to identify the focus area within the visual field. Although the concept is straightforward, the techniques and analyses can be complex. In InfoVis contexts, eye activity provides direct indicators of how users allocate attention across visual elements, making it a valuable signal for understanding interaction patterns and cognitive effort.

Eye-tracking data are typically gathered using either screen-based or wearable eye trackers connected to a computer. Modern eye-tracking systems usually consist of two main components: an infrared light source and a camera. The infrared light source targets the eye, and the camera tracks the reflection along with distinct ocular features such as the pupil (Joseph and Murugesh, 2020). As eye-tracking data are primarily used to study eye movements and determine gaze direction. Below is a summary of the most common features representing eye and pupil behavior:

**Fixations:** Maintaining eye gaze on a single point. This is due to the fovea, which provides the clearest vision (Rehman et al., 2023). Common fixation metrics include: number of fixations, total fixations, fixation duration, etc. (Bylinskii et al., 2017).

**Saccades:** Quick shifts of the eyes between fixation points (Kowler, 2011). Common saccadic features include: number of saccades, saccadic velocity/amplitude, saccade rate, and duration (Rucci and Poletti, 2015).

**Pupil variation:** Constriction and dilation of pupil muscles. It reflects autonomic involuntary activity and is linked to emotional, cognitive, or sexual arousal (Bradley et al., 2008).

**Eye blinks:** Semi-involuntary action of quickly closing and reopening the eyelid. The most popular metric is the blink rate (BR), measuring amount of blinks per minute (Ousler III et al., 2008).

Therefore, commonly used features include fixation duration, saccade amplitude, blink rate, and pupil dilation, which reflect how users process and navigate visual information. Recent work has further shown that eye activity provides meaningful implicit feedback signals in high-level decision-making tasks, such as evaluating model-generated responses (Lopez-Cardona et al., 2025b).

Furthermore, complementary features such as saliency coverage (Walter and Bex, 2022) and saliency entropy (Cui et al., 2024) reliably increase under higher task difficulty, reflecting more extensive visual exploration. By doing so, these properties make eye activity a highly informative modality for monitoring cognitive state during interaction with visualizations. To visualize these metrics, heatmaps and gaze plots are commonly used. As a result, eye-tracking can quantify and visualize intuitively features of cognitive workload, a key factor in research and design. This was demonstrated in recent work to study cognitive processing during complex evaluation tasks, where the authors demonstrated its relevance beyond InfoVis and into settings such as human alignment of large language models (Lopez-Cardona et al., 2025c).

## 4 Cognitive processes

The significance of investigating mental fatigue, mental workload, stress, and mind wandering in cognitive processes is well-established, and supported by numerous studies and reviews that will be discussed across this section. Their prioritization in cognitive research stems from their impact on human performance and overall cognitive function in InfoVis environments (Ismail and Karwowski, 2020). Understanding these cognitive processes is crucial for optimizing interactions and designing more effective information visualization systems that can adapt to users' cognitive states. In particular, these cognitive states directly influence how users explore, interpret, and act upon visual information, making them central to the design of neuroadaptive InfoVis systems.

### 4.1 Mental fatigue

Mental fatigue refers to a cognitive state characterized by a decreased alertness, impaired performance and diminished judgement (Nguyen et al., 2017; Bendak and Rashid, 2020). Mental fatigue is usually caused by prolonged cognitive activity (Van Cutsem et al., 2017). Apart from the lack of sleep or rest and extensive cognitive activity, other important factors that lead to mental fatigue are disruption of circadian rhythms (Boivin and Boudreau, 2014), substance abuse (Bootzin and Stevens, 2005), and heat exposure (Van Cutsem et al., 2017).

Mental fatigue is one of the aspects of cognitive state widely studied in neuroergonomics as it accounts for a large proportion of road traffic accidents and deaths, as well as accidents in several sectors such as manufacturing, medicine, mining and aerospace. Based on federal data, about 15%–33% of fatal road accidents are believed to be caused by drowsy driving (Maia et al., 2013), while the cost of accidents due to drowsy driving is estimated to be between $139 billion and $153 billion in the United States, and between 43 billion and 337 billion in Europe (Léger et al., 2019). In addition, mental fatigue is a safety concern in aviation operations and can impact the psychological and physical health of crew members (Bendak and Rashid, 2020). The negative consequences of mental fatigue is also observed in clinical settings, where misdiagnoses in radiology due to mental fatigue can have a detrimental impact on patients' health outcome and lead to medical malpractice (Nihashi et al., 2019). From an InfoVis perspective, mental fatigue reduces users' ability to process visual information, identify relevant patterns, and maintain situational awareness during complex analytic tasks. Understanding how mental fatigue manifests during interaction with visualizations is therefore essential for the development of neuroadaptive InfoVis systems. As such, research efforts focus on the development of methods for detecting mental fatigue using a variety of neurophysiological measures, including HR (Fujiwara et al., 2019), EDA (Posada-Quintero et al., 2017), eye-tracking (Nguyen et al., 2015), EEG (Cao et al., 2014), and fNIRS (Nguyen et al., 2017; Tanveer et al., 2019a), measurements that are possible to collect outside the laboratory, in an ecologically valid setting. Finally, although mental fatigue and drowsiness are distinct constructs, the neurophysiological literature often reports overlapping biomarkers and experimental paradigms for both states (Borghini et al., 2014). For consistency throughout this review, we include findings from studies on drowsiness when they reflect declines in alertness or cognitive performance that are also characteristic of mental fatigue.

#### 4.1.1 Assessing mental fatigue with EEG

A widely used measure for deriving ongoing vigilance levels from EEG data is the alpha slow-wave index, which is defined as the power in the alpha band divided by the power in the delta and theta bands; i.e., α/(δ+θ) (Horovitz et al., 2008; Wong et al., 2013). Discrete vigilance stages from alertness to sleep onset have also been defined based on the levels of alpha power and its spatial distribution, with relatively high levels of anterior and posterior slow waves (i.e., delta and theta band) indicating tiredness (Olbrich et al., 2009). Furthermore, other works have proposed the increases in alpha power or the (θ+α)/β and α/β ratios as measures for detecting decline in alertness or increasing levels of fatigue (Cao et al., 2014; Kqthner et al., 2014).

Some inconsistencies found in the literature may partly stem from differences in methodology, such as the strategy used for preprocessing the EEG data (Craig et al., 2012) or the technique used for computing the PSD. In addition, inconsistencies could also be due to the experimental paradigms. For example, in cases where the subject is in a resting-state or is performing a task that is not mentally demanding (e.g., pressing a button at the presence of an auditory stimulus), fluctuations in mental fatigue may appear as shifts of power from the alpha band to lower frequency bands, whereas in cases where the subject is doing a task that is relatively demanding (e.g., during simulated driving), mental fatigue is reflected as a shift of power from the beta to the alpha power (Eoh et al., 2005).

Some studies have shown that alpha spindles, which consist of short (0.5–2 sec) bursts of alpha activity, are superior to alpha power in detecting fluctuations in fatigue (Schmidt et al., 2009; Simon et al., 2011). Specifically, higher alpha spindle rates and longer alpha spindle durations have been observed during periods of high mental fatigue. Additionally, Sonnleitner et al. (2012) demonstrated that alpha spindle parameters are sensitive to shift in attention, providing also evidence that alpha spindles may reflect inhibition of visual information processing.

Another approach for detecting fatigue levels is the examination of Event Related Potentials (ERPs) which refer to average responses, time-locked to the onset of a series of identical or very similar stimuli. Through the process of averaging, the background noise is suppressed allowing consistent patterns of EEG activity to be revealed. ERPs consist of positive and negative deflections in the voltage which can be studied based on the amplitude and latency of each peak. The peaks are labeled based on their polarity (positive or negative) and order (e.g., P1, N1, P2, and N2) or approximate time in milliseconds (e.g., N100, P200, and P300). A common experimental paradigm, in the context of fatigue detection, is the auditory oddball where the participants are presented a series of frequent and infrequent (odd) tones, and are instructed to respond quickly to the infrequent tones by pressing a button. In this paradigm, a larger P300 amplitude (i.e., positive peak that occurs around 300 ms after the stimulus onset) is observed with the infrequent tones. Samima et al. (2017) found that high fatigue was associated with decreased amplitude of P300 during the auditory oddball. On the other hand, Trejo et al. (2005), using a mental arithmetic task, found the P200 amplitude to be affected by fatigue as assessed by the time on task, whereas the N100 and P300 did not show any significant change. The use of ERP analysis for fatigue detection has been somewhat limited in the area of cognitive performance assessment as, by definition, ERP analysis requires the presence of discrete events or stimuli and, thus, it cannot be applied in studies where participants are engaged in an ongoing task (e.g., air-traffic control).

#### 4.1.2 Assessing mental fatigue with fNIRS

Several fNIRS studies have demonstrated a strong association of mental fatigue with activity in the prefrontal cortex. However, the brain laterality and direction of brain activity (e.g., increase/decrease in HbO2 levels) related to fatigue differed across studies. For example, Liu (2014) reported increased activity in the left prefrontal cortex during periods of fatigue, whereas subsequent studies found an increase in the right prefrontal cortex during similar conditions (Khan and Hong, 2015; Tanveer et al., 2019a). Moreover, with regards to direction of activity, while some studies found the HbO levels in prefrontal cortex to be positively correlated with fatigue (Liu, 2014; Khan and Hong, 2015; Khan et al., 2019), other studies found these measures to be negatively correlated (Nguyen et al., 2017; Nihashi et al., 2019).

Features obtained with fNIRS related to regional activity (e.g., moving average of HbO2 levels) and connectivity between pairs of regions (wavelet coherence) have been shown to greatly benefit Machine Learning (ML) and DL approaches in detecting mental fatigue, with accuracies ranging from 67%–99% (Ahn et al., 2016; Dehais et al., 2018; Khan et al., 2019; Tanveer et al., 2019a). These accuracy values were obtained through supervised classification approaches, typically using k-fold or leave-one-subject-out cross-validation to distinguish fatigued vs. alert states. Although similar tasks were employed in these studies, such as driving and flight simulation, different strategies were used to detect periods with fatigue. These range from self-reported assessment (Liu, 2014) and behavioral performance [e.g., traffic violation during driving task (Khan and Hong, 2015)] to neurophysiological measures [EEG beta activity, eye closure, and face expression (Nguyen et al., 2017; Tanveer et al., 2019a)], with each strategy having its own limitations for accurate detection. As such, it is still unclear which fNIRS-derived features are more informative in terms of mental fatigue detection. Most features though seem to lead to improved detection, as compared to detection based solely on EEG recordings (Ahn et al., 2016; Dehais et al., 2018; Khan et al., 2019).

#### 4.1.3 Assessing mental fatigue with HRV

Lower alertness and mental fatigue have been associated with increases in low-frequency power of HRV, which may suggest a shift toward sympathetic dominance during periods of reduced cognitive readiness (Chua et al., 2008; Tran et al., 2009; Zhang and Yu, 2010; Goffeng et al., 2019; Qin et al., 2021). In some studies, lower alertness and mental fatigue have also been linked to increased LF/HF and decreased HF, which may suggest a shift of the predominant activity of the autonomic nervous system from parasympathetic to sympathetic activity. Moreover, Goffeng et al. (2019) showed that subjects with shorter duration of sleep before work present lower RMSSD and SDNN, which can be interpreted as individuals who are less alert are characterized by decreased parasympathetic activity. However, some prior works have found contradicting results, such as decreases in the LF/HF measure during mental fatigue (Vicente et al., 2016) and increases during times with increased alertness. Therefore, further research is needed to evaluate the reliability of HRV measures for detecting mental fatigue.

#### 4.1.4 Assessing mental fatigue with EDA

With regard to state of mental fatigue, prior research has demonstrated the usefulness of the EDA measure in predicting such state in different contexts. For example, Williamson et al. (2020) used tonic and phasic features of EDA, in combination with speech and facial features, to detect states of mental fatigue in soldiers after two hours of cognitive effort. The use of EDA features provided high performance in predicting fatigue levels (AUC ≃ 99%), while the fusion of features across these different modalities produced even higher detection accuracy (AUC = 99%). These AUC values correspond to binary classifiers evaluated with k-fold cross-validation. Zhang et al. (2018) collected EDA from healthy subjects for 6 hours per day for 6 weeks, in conjunction with other physiological measures (cardiac activity and skin temperature) and detected participant-reported fatigue with an accuracy of 82.9%. This accuracy was obtained by training a deep neural model and evaluating it on a held-out test subset of the recordings. As a result, it highlights the stability of EDA-based fatigue markers over extended time scales.

Finally, in the study conducted by Amidei et al. (2023), an accuracy of 89.4% in detecting driver's mental fatigue was achieved with EDA. This accuracy reflects binary driver-fatigue classification evaluated using k-fold cross-validation, demonstrating its robustness across different tasks and application domains.

#### 4.1.5 Assessing mental fatigue with eye-tracking

A substantial body of research has explored how eye-tracking metrics evolve during cognitive tasks, aiming to identify sensitive indicators of increasing mental fatigue. Although the link between pupil metrics and mental fatigue has been studied for a long time recent advancements in eye-tracking devices have significantly expanded this field of research. Among the various studied metrics, pupil diameter and blinking (Benedetto et al., 2011) are among the most easily observable visual behaviors. Numerous studies have demonstrated that increased fatigue leads to longer and more frequent blinks (Benedetto et al., 2011). Other research has identified metrics such as blink velocity and interval as related to heightened mental fatigue (Di Stasi et al., 2012).

Saccadic metrics have also been associated with mental fatigue (Di Stasi et al., 2013). Prior work has explored the validity and sensitivity of saccadic metrics, including saccade velocity, duration, and amplitude, as indicators of an individual's fatigue (Di Stasi et al., 2012, 2013). Saccade magnitude has been shown to decrease under fatigued conditions, alongside reductions in pupil diameter and increases in blink duration (Hu, 2022).

Several studies have utilized these features to classify gaze data according to different levels of mental fatigue. For example, the model presented by Li and colleagues demonstrated an accuracy of 87.10% in detecting fatigue levels (Li et al., 2022), while Yamada and Kobayashi (2018) and Marshall (2007) reported accuracies of 91% and 92%, respectively. These accuracy values reflect binary fatigue-detection models evaluated using either train/test splits or cross-validation, depending on the methodology reported in each study. Furthermore, these results collectively highlight the discriminative power of eye-tracking features for identifying fatigue-related changes in visual behavior.

#### 4.1.6 InfoVis applications of mental fatigue

Mental fatigue has direct implications for users' ability to engage with information visualizations, as many visual analytic tasks rely heavily on sustained attention, stable gaze behavior, and efficient visual search. Eye-tracking studies in visualization contexts have shown that users acquire most of the information necessary for task performance within the first moments of interaction, and that both the speed and structure of visual exploration are sensitive to changes in cognitive effort (Spiller et al., 2021; Karolus et al., 2017). Under fatigue, these gaze patterns become less efficient, characterized by longer fixations, reduced saccade amplitude, and increased blink frequency, indicating a decline in the user's capacity to extract structure and meaning from complex visual scenes (Di Stasi et al., 2012, 2013; Hu, 2022; Yamada and Kobayashi, 2018; Marshall, 2007).

Because visualization tasks often require iterative scanning, comparison, and integration of multiple visual elements, decrements in attentional resources can disproportionately affect performance compared to tasks that rely less on visual processing. For example, fatigue-related increases in blink duration or reductions in pupil diameter have been linked to slower visual search and higher error rates during decision-making tasks (Sciaraffa et al., 2021), suggesting that mental fatigue weakens the perceptual and attentional mechanisms that support effective visualization use.

Across studies involving sustained attention and high-demand visual tasks, physiological markers of mental fatigue have been associated with reduced accuracy, slower responses, and greater variability in user performance (Eoh et al., 2005; Trejo et al., 2005; Samima et al., 2017). These effects imply that users may struggle to maintain stable performance when visualizations require continuous monitoring, pattern detection, or rapid judgment.

Together, these findings show that mental fatigue alters the perceptual, attentional, and decision-making processes that underpin interaction with information visualizations. As such, fatigue-related physiological markers (in particular those derived from gaze behavior) offer a relevant foundation for developing InfoVis systems that can detect when users' cognitive resources are declining and adapt accordingly.

### 4.2 Mental workload

Mental workload, also referred to as cognitive workload, refers to the portion of an individual's mental capacity or resources that are required by task demands. Previous research has shown that mental workload presents an inverted U-shape relationship with performance, where moderate levels of workload yield high performance levels, while low and high levels of workload are associated with impaired performance (Bruggen, 2015; Yerkes et al., 1908). Moderate levels of workload are believed to stimulate the operator and thus keeping them focused on the task. On the contrary, simple tasks that are not mentally demanding often lead to boredom and to a state of low vigilance and attention levels (underload condition), which increases the occurrence of errors. Similarly, complex and demanding tasks, either in terms of number of tasks or task difficulty, can lead the operator to an overload condition that is also characterized by an increased occurrence of errors. Specifically, in safety-critical applications, such as in aviation operations, a large portion of accidents (airplane crashes, critical commercial systems failures, etc.) that result in loss of lives and money (Aricó et al., 2017) is attributed to high mental workload. It has been suggested that high mental workload can lead to a significant depletion of the cognitive resources of the operator, which can compromise situation awareness (Borghini et al., 2014). Situation awareness refers to how well an operator perceives various environmental elements, as well as their projection in time and space. In cases where operators need to make critical choices, poor situation awareness that is often caused by increased levels of mental workload, may result in catastrophic outcomes.

Given the crucial impact of mental workload on human performance, safety-critical systems are designed to balance the information provided to operators—concerning engine and monitoring systems (e.g., through the dashboard of modern cars)—and the need to remain attentive to the external environment, such as watching the road while driving (Borghini et al., 2014). However, although the performance of an individual depends on their mental workload, assessing and comparing the mental workload across individuals based on their performance can be misleading. Mental workload depends on several factors like the subjective experience of the operator with the task, the circumstances under which the task is performed, as well as individual differences. Consequently, even when two operators demonstrate comparable performance on a given task, their perceived mental workload may differ significantly. To address this challenge, researchers have been employing neurophysiological indicators, obtained with EEG (Zhou et al., 2022) and fNIRS (Midha et al., 2021). These signals provide a continuous and objective assessment of mental workload, which is of great value as it can enable real-time adaptation, ensuring that mental workload remains within desired boundaries.

#### 4.2.1 Assessing mental workload with EEG

Researchers have been investigating the potentials of EEG for assessing the levels of mental workload for more than two decades (Ismail and Karwowski, 2020). Early studies found that as task difficulty increases, theta activity in the frontal midline areas also increases while alpha activity in parietal areas decreases, indicating the potential of alpha and theta frequency bands for assessing mental workload. In a subsequent study, the Task Load Index (TLI), defined as the ratio of frontal (Fz) theta to parietal (Pz) alpha power (θ/α), was found to be more sensitive to mental workload as compared to the associated individual bands (Holm et al., 2009). However, some studies have found no association of alpha and theta activity with mental workload (Kqthner et al., 2014), whereas others suggested that the sensitivities of EEG parameters depend on the task (Gomarus et al., 2006; Lei and Roetting, 2011). For example, (Gomarus et al. 2006) observed decreased alpha power during the high load condition of a memory encoding task and no significant change in theta power, whereas significant increase in theta power and no change in alpha power was observed in the high load condition of a recognition task.

Another widely used measure of mental workload is the engagement index defined as the ratio of beta to alpha and theta power β/(α+θ) pooled over four electrode sites (Cz, P3, Pz, and P4). It remains unclear how the engagement index compares to the task load index; however, data from a small cohort of four participants suggest that the latter may provide superior performance (Hockey et al., 2009). Intriguingly, the findings of some studies that sought to examine brain responses to variations in task demand led to conclusions inconsistent with the task load and engagement indices. For example, the study by Murata (2005) found elevated theta, alpha and beta activity in high workload periods.

The P300 amplitude which, as mentioned earlier, is often used for detection of fatigue levels, seems to be strongly linked to mental workload as well. Specifically, a decreased P300 amplitude is typically found in the parietal area in periods of high cognitive tasks (Gomarus et al., 2006; Allison and Polich, 2008; Holm et al., 2009; Miller et al., 2011; Kqthner et al., 2014; Dehais et al., 2019). An inverse relationship between task load and ERP components has also been reported for the N100 and P200, although the effect for the P300 amplitude seems to be the most prominent (Allison and Polich, 2008; Miller et al., 2011). As a reminder, to extract the ERP components, the participants need to be exposed to a series of identical or similar stimuli, and to measure the levels of mental workload, the auditory oddball task or a modification of it is typically exploited. Based on the findings of Allison and Polich (2008), the association of ERP components with variations in task difficulty is stronger when subjects ignore the tones of the oddball task than when they count the tones. This finding suggests that the ERP paradigm needed for probing mental workload can be less distracting than in conventional oddball tasks, so that the participants focus exclusively on a given task.

#### 4.2.2 Assessing mental workload with fNIRS

Fluctuations in the haemodynamic activity of the prefrontal cortex have also been associated with variations in task difficulty for a range of tasks (Gateau et al., 2015), demonstrating the potential of fNIRS measurements for assessing mental workload. The most common finding has been an elevation in the HbO2 levels of the prefrontal cortex with increased levels of task difficulty, which has been reported by several groups examining piloting tasks (Durantin et al., 2014; Harrison et al., 2014; Gateau et al., 2015) or the n-back task (Fishburn et al., 2014). Increases in HbO2 and decreases in HbR have also been linked to increased workload demands in reading tasks (Midha et al., 2021), as well as to display formats (i.e., bar graph and pie chart) and web form layouts that were perceived by users as more mentally demanding (Peck et al., 2013; Lukanov et al., 2016). In some studies examining expertise development, participants were asked to perform and repeat for a couple of days some basic working memory tasks or piloting tasks for which they did not have prior experience (Ayaz et al., 2012). Interestingly, apart from the improvement in performance observed over the course of days, there was also a significant decrease in prefrontal activity, indicating a reduction in mental effort required from participants due to the acquisition of the necessary skills.

Despite the accumulating evidence for the role of prefrontal activity in mental processes, there are tasks such as writing tasks or a supervisory control task for which previous studies were not able to find any link between fNIRS measurements and changes in task difficulty (Boyer et al., 2015; Midha et al., 2021). It is still unclear, however, if these negative findings are due to the nature of the task examined or due to suboptimal choices with regards to the collection and analysis of fNIRS measurements. As it has been shown by Mandrick et al. (2013), the standard approach for examining the effect of a stimulus on fNIRS measurements based on the mean magnitude in the pre-stimulus and post-stimulus period, is somewhat limited in terms of detecting variations in task difficulty. Instead, the authors propose measuring the slope of the fNIRS signal over the entire stimulus period through linear regression in order to improve sensitivity to variations of difficulty. Moreover, recent studies have indicated that connectivity features derived from fNIRS recordings (e.g., wavelet coherence in fNIRS activity of two regions) can substantially improve the assessment of mental effort as compared to using only information related to regional activity (Fishburn et al., 2014). Therefore, further research is needed to understand how to best probe variations in mental effort based on fNIRS.

#### 4.2.3 Assessing mental workload with HRV

The use of HRV measures for assessing mental workload has been extensively examined using a range of tasks, with the most common experimental paradigms being driving (Stuiver et al., 2014; Heine et al., 2017) and aviation tasks (Mansikka et al., 2019; Radüntz et al., 2021). A common finding has been that increases in the levels of task difficulty are accompanied by increases in HR (or equivalently decreases in beat-to-beat intervals) and decreases in HRV measures (Engstrm et al., 2005; De Rivecourt et al., 2008; Durantin et al., 2014). These physiological responses are consistent with an increase in sympathetic activity which may be necessary for dealing with more demanding tasks. In a recent study, it was also shown that HR and HRV measures could distinguish varying levels of task difficulty across different segments of an aviation task, even when the performance of the participants was similar (Mansikka et al., 2019). However, it should be noted that there were several studies in which HRV measures could not distinguish different levels of task difficulty, and were only able to discriminate task from rest. In addition, some studies have found contradicting results, such as increases in the HRV measures LF/HR and SDNN during periods with high mental workload as compared to periods with simple tasks (Skibniewski et al., 2015). Given this, it is still unclear whether HR and its derivatives can be used, without any other physiological signal, for assessing the mental workload of an individual. Based on recent work (Radüntz et al., 2021), changes in mental workload are reflected immediately on HR traces, whereas responses in HRV measures, including the LF/HF ratio, may lag up to 6 minutes with respect to the changes in mental workload. Therefore, to improve the reliability of HRV measures for detecting variations in mental workload, new strategies need to be developed that take into consideration the measures' inherent timescales.

#### 4.2.4 Assessing mental workload with EDA

EDA has also shown great potential for the detection of mental workload. For example, the study by Meteier et al. (2021) combined EDA, cardiac activity, and respiration signals to classify the level of mental workload of drivers with an accuracy of 95%. This accuracy was obtained with a binary classifier distinguishing high vs. low workload, evaluated using 10-fold k-fold cross-validation on the physiological features. Used alone, however, the predictive potential of EDA for determining mental workload seems more limited. In this regard, Romine et al. (2022) used the EDA signal to determine the mental effort experienced by a single participant in daily activities, including attending class and leisure activities, among others. Data were collected for over 90 h, and the prediction of mental effort provided considerable accuracy (AUC = 63%, F1 = 63%), but was still far from optimal. These performance values were obtained using a supervised classifier trained on windowed EDA features and evaluated with cross-validation on the participant's own time-series data.

#### 4.2.5 Assessing mental workload with eye-tracking

The assessment of mental workload is one of the most extensively studied cognitive features related to eye-tracking metrics. Eye-tracking systems offer numerous opportunities for studying user behaviors in a low-cost, non-intrusive, and ecologically valid manner. This association is not only relevant for research and design but also holds significant interest for fields such as advertising, entertainment, and interface design, where understanding user behavior is crucial.

Pupil dilation is a key indicator of cognitive load in eye-tracking research. This involuntary reflex causes the pupil to change in diameter, ranging from 0.15 cm to more than 0.8 cm (Joseph and Murugesh, 2020). However, multiple factors can trigger it, such as light reflex, changes in illumination, and errors due to gaze angles. Additionally, human pupils dilate for various cognitive/emotional reasons, including memory recall, cognitive effort, excitement, and pain. Consequently, to assess changes in cognitive load across different tasks, researchers must carefully analyze the average percentage change in pupil size and identify the specific cause of the pupil response (Joseph and Murugesh, 2020). In other words, careful interpretation is required to separate workload-related dilation from other sources of pupil variation.

Previous studies have shown that average fixation duration is significantly negatively correlated with the level of cognitive load in both simulated flight (De Rivecourt et al., 2008) and driving tasks (Foy and Chapman, 2018). Additionally, blink rate, microsaccade rate, and blink duration all appear to decrease as cognitive load increases (Skaramagkas et al., 2021). It is essential to note that cognitive workload is heavily influenced by the user's level of expertise. While novices have been found to exhibit longer fixation durations compared to experts in various tasks, such as surgical settings (Menekse Dalveren and Cagiltay, 2018) and chess playing (Sheridan and Reingold, 2017), no significant difference in fixation duration was observed between expert and novice map users (Keskin et al., 2020). Thus, considering the level of experience of users is crucial when studying the relationship between eye-tracking metrics and mental workload. In conclusion, these findings suggest that eye-movement indicators related to cognitive workload are influenced by both task characteristics and user expertise; however, these effects are not uniform across all metrics, and some measures (e.g., fixation duration) may show no significant differences between expert and novice users in specific tasks.

#### 4.2.6 InfoVis applications for mental workload

Mental workload has a direct influence on the cognitive processes that underpin effective interaction with information visualizations. As task demands increase, users allocate more attentional and working-memory resources to maintain performance, which affects how they scan, interpret, and integrate visual information. Studies in visually demanding environments such as driving, aviation, and monitoring tasks consistently show that higher workload leads to slower visual search, reduced fixation dispersion, and more narrowly focused gaze patterns, indicating that users rely on more selective processing when their cognitive capacity is constrained (De Rivecourt et al., 2008; Foy and Chapman, 2018; Skaramagkas et al., 2021).

In visualization contexts, increases in cognitive load can reduce the efficiency with which users extract relationships, compare values, or detect anomalies, particularly when displays contain dense or rapidly changing information. Evidence from tasks involving graph interpretation, map reading, and complex spatial displays suggests that elevated workload is associated with longer fixation durations and reduced exploration breadth, reflecting a shift toward more effortful processing and a decreased ability to manage competing visual elements (Keskin et al., 2020; Menekse Dalveren and Cagiltay, 2018).

User expertise also modulates this relationship: while experienced users can distribute attention more effectively across a visualization, novices often show longer or less structured gaze patterns under high workload, which can increase cognitive demands and lead to performance degradation in complex visual analytic tasks (Sheridan and Reingold, 2017).

Together, these findings highlight that mental workload alters the perceptual, attentional, and memory-related mechanisms needed to navigate and reason with information visualizations. Understanding how workload manifests in visual behavior provides a basis for developing InfoVis systems that better support users during periods of high cognitive demand; for example, by adapting visual complexity, modulating update rates, or highlighting information relevant to the user's current cognitive state.

### 4.3 Stress

Several definitions have been proposed over the years for the term “stress.” In this context, stress refers to a psychophysiological response to perceived demands or threats (often described as a “fight-or-flight” response) that can affect attention, decision-making, and performance. Stress is commonly described as acute, episodic, or chronic, depending on duration and recurrence.

Acute stress is induced by a brief stressor, and is characterized by an increase in secretion of cortisol along with transient changes in physiological processes (e.g., increase in HR). In several cases, these brief stressors occur repeatedly in the working environment, leading to a stress type termed episodic stress. This type of stress is not continuous and eventually ceases at the end of a critical period. Chronic stress, on the other hand, results from persistent stress over a long period of time and can lead to deterioration of physical and mental health (Baevskii et al., 2009).

In order to prevent chronic stress, which is the most harmful type of stress, it is important to be able to detect acute or episodic stress at an early stage and take appropriate measures, such as treating the underlying causes or utilizing stress management techniques. Over the last decades, a wide range of approaches have been explored for providing non-invasive and automatic detection of stress using physiological (EEG, fNIRS, HR, blood pressure, etc.) and physical (facial expressions, pupil diameter, voice intonation, etc.) information (Alberdi et al., 2016; Rastgoo et al., 2019; Giannakakis et al., 2022). The aforementioned techniques are based on a cascade of physiological processes that are triggered when one perceives a threat and are collectively referred to as the stress or fight or flight response. Some of the main responses are the increase in cardiac and breathing rate, as well as the dilation of the pupils. The stress response is coordinated by the sympathetic nervous system and is considered essential for surviving in threatening situations. Note that as suggested by several studies (Dhabhar, 2014), under certain conditions, stress can have positive effects on one's performance as well as immune function and, thus, there is often the distinction between positive (“eustress”) and negative (“distress”) stress. However, in the field of neuroergonomics, the term “stress” typically refers only to negative stress and, thus, we follow herein the same practice.

#### 4.3.1 Assessing stress with EEG

With regards to stress assessment, frontal alpha asymmetry has been the most widely used measure in the field of neuroscience. Increased relative alpha activity in right anterior and posterior areas was found to be associated with the presence of anxiety as well as with the risk for the development of anxiety and depressive symptoms in the following year (Blackhart et al., 2006). Further evidence supporting the role of stress in frontal alpha asymmetry comes from the study of Lopez-Duran et al. (2012) where the authors show that children that had experienced high levels of episodic and chronic stress present greater relative right frontal activation than their peers with less exposure to stressful life events. Moreover, increased levels of frontal alpha asymmetry have been observed during stressful periods (Lewis et al., 2007; Lopez-Duran et al., 2012; Giannakakis et al., 2015).

Although frontal alpha asymmetry is likely the most commonly used measure for stress assessment, recent studies have found a few other measures that seem to be more sensitive to stress levels. One of those measures is the prefrontal relative gamma power, defined as the ratio between gamma (25–45 Hz) and slow rhythms (4–13 Hz), which has been shown to increase at periods with high stress and to be strongly correlated with variations in HR (Minguillon et al., 2016). Alonso et al. (2015) examined a variety of EEG measures in the context of stress assessment and, although the authors did find a shift of relative frontal alpha activity from the left to the right hemisphere following stress tests, the changes in levels of frontal alpha asymmetry were not statistically significant. In contrast, the authors reported several other measures, including the EEG power in the high-alpha and high-beta bands, approximate entropy, and interhemispheric non-linear couplings, to better discriminate the pre- and post-stressor conditions. Furthermore, reduced alpha and gamma activity have been observed in the entire cortex during stress conditions (Tran et al., 2007) and in elderly subjects characterized with high levels of stress (Marshall et al., 2015), whereas increases in theta and alpha power have been observed during Zen meditation, a mental activity associated with stress reduction (Tetsuya Takahashi et al., 2005).

#### 4.3.2 Assessing stress with fNIRS

Currently, there is only a small number of studies that have examined the neural correlates of stress with fNIRS with somewhat inconclusive results. One of the first studies was by Rosenbaum et al. (2018) who used the Trier Social Stress Test (TSST) to induce stress in their cohort. The authors found the induced stress to be linked to increased levels of HbO2 in the dorsolateral prefrontal cortex, the inferior frontal gyrus and the superior parietal cortex. Similarly, Schaal et al. (2019) showed that the HbO2 levels in the dorsolateral prefrontal cortex, as well as in the orbitofrontal cortex, can increase in response to stress induced with the Maastricht Acute Stress Test (MAST). With respect to functional connectivity, a subsequent study that considered a simulated pipe maintenance task found enhanced interhemispheric connections during the stressful conditions, and particularly enhanced connections between the primary motor areas of the left and right hemispheres (Shi et al., 2020). Different findings have been provided by another work that utilized the Montreal Imaging Stress Task (MIST) and the Stroop Color-Word Test (SCWT) as the stressors (Al-Shargie et al., 2015, 2016, 2022). Specifically, a decrease in prefrontal oxygenations was observed during the MIST task as compared to the control task (Al-Shargie et al., 2016, 2015), while an increase in connectivity between the ventrolateral and dorsolateral prefrontal cortex was observed during the stressful condition of the SCWT (Al-Shargie et al., 2022).

#### 4.3.3 Assessing stress with HRV

Previous work has examined the relationship of HRV with a variety of stressors, such as those that occur naturally throughout the day or through different periods (e.g., during a semester at the university vs before examination period), as well as those induced by suitable tests in the laboratory (e.g., the trier social stress test) (Kim et al., 2018). Similar to the approaches for detecting mental fatighe and mental workload, HRV changes associated with a shift from parasympathetic to sympathetic activity have been shown to track variations in stress (Giannakakis et al., 2022). The majority of studies have found periods with stress to be accompanied by increases in HR (Dimitriev et al., 2008; Clays et al., 2011; McDuff et al., 2014; Pereira et al., 2017) and levels of LF/HF ratio (Dimitriev et al., 2008; Pereira et al., 2017), as well as decreases in the SDNN (Tharion et al., 2009; Taelman et al., 2011; Pereira et al., 2017), RMSSD (Taelman et al., 2011; Acerbi et al., 2017; Pereira et al., 2017) and HF component of HRV (Dimitriev et al., 2008; Pereira et al., 2017). Pereira et al. (2017) investigated the sensitivity of a range of time-domain and frequency-domain HRV measures and found the mean RR interval (or equivalently the mean HR) to yield the best performance. In addition, they examined time window lengths ranging from 50 to 220 sec, and found the shortest one (i.e. 50 sec) to be adequate for monitoring stress levels. However, note that, even though the majority of studies have reported similar effects of stress on cardiac rhythms, studies with null results or inconsistent findings are not missing from the literature (Schubert et al., 2009; McDuff et al., 2014).

#### 4.3.4 Assessing stress with EDA

One of the areas in which EDA may have the greatest potential is in the detection of stress, as shown by several studies (D'Amelio et al., 2025). For example, Liu and Du (2018) achieved a recognition rate of 81% in the detection of driver stress states using only the EDA signal measured on participants' feet (with this value obtained using a supervised classifier evaluated through cross-validation); while Greco et al. (2023), using a Support Vector Machine (SVM) for state classification, were able to classify the state of the participants (stress/non-stress) with an accuracy of 94.6%, based on a binary stress classification model assessed using k-fold cross-validation. Furthermore, the results by Nardelli et al. (2022) suggest that the complexity of the EDA signal (and, in particular, of its two—tonic and phasic—components) can be used to discriminate not only between stress and resting states but also between different types of stress, with a physical or cognitive origin. These findings highlight the richness of EDA features for characterizing multiple stress-related responses.

#### 4.3.5 Assessing stress with eye-tracking

The relationship between stress and eye-tracking metrics has been extensively studied, particularly as stress becomes an increasingly prevalent issue in modern society. For example, participants with social anxiety have shown no initial orienting bias under stressful situations and are more likely to fixate on angry faces, with longer fixation duration compared to those without anxiety (Liang et al., 2017). In general, the rate of spontaneous eye blinks increases, and blinking patterns undergo notable changes during stress or other emotional states (Giannakakis et al., 2015).

The connection between eye blink frequency and stress levels has been established, with both artificially induced emotional responses (e.g., through billboards) and more natural emotional responses (e.g., simulated car crashes) leading to a temporary rise in eye blink frequency (Haak et al., 2009). Another relevant feature associated with stress is pupil size, which has been robustly used as an index of stress over the years (Zhai and Barreto, 2006; Wang et al., 2018). Among individuals with elevated stress levels, the pupil response tends to be more pronounced when visual stimuli with negative valence are presented (Kimble et al., 2010). Increased anxiety levels correlate with larger pupil size, showing significant variations during expressions of contempt and surprise (Wang and Yue, 2011). The distribution of gaze points is also believed to be influenced by arousal and stress (Laretzaki et al., 2011). When classifying between stress and stress-free conditions, the addition of eye-tracking features has been shown to enhance EDA analysis, improving detection accuracy by 3.72% in a Stroop task and 0.45% in a mathematical task on average (Yousefi et al., 2022), with these improvements calculated from binary stress classifiers evaluated using k-fold cross-validation across participants. This suggests that combining gaze behaviors with peripheral physiological measures can strengthen stress-detection performance.

#### 4.3.6 InfoVis applications for stress

Stress influences several perceptual and cognitive mechanisms that are central to interacting with information visualizations. Under stress, users tend to narrow their attentional focus, show stronger orienting responses to threatening or negatively valenced stimuli, and display less flexible visual exploration patterns, as reflected in longer or biased fixations toward emotionally salient items (Liang et al., 2017; Laretzaki et al., 2011). Such changes can reduce a user's ability to scan a visualization broadly, integrate information across multiple visual elements, or evaluate alternative interpretations during analytic reasoning.

Eye-tracking studies on stress further show increased blink rates, altered blink patterns, and enlarged pupil responses during emotionally or cognitively demanding situations (Giannakakis et al., 2015; Haak et al., 2009; Zhai and Barreto, 2006; Wang et al., 2018). These behaviors indicate heightened arousal and can serve as reliable indicators of when users may be experiencing difficulty processing complex or ambiguous displays. When combined with peripheral physiological signals such as EDA, the inclusion of gaze-derived features has been shown to improve classification of stress states (Yousefi et al., 2022), highlighting the potential of multimodal sensing to support visualization systems that adapt to users' emotional and cognitive conditions.

In high-stakes or time-critical visualization environments, stress can impair decision-making, reduce working-memory availability, and increase the likelihood of over-focusing on specific details at the expense of global patterns. These effects underscore the value of incorporating stress-sensitive physiological monitoring into adaptive InfoVis systems. Doing so may enable interfaces that modulate visual complexity, adjust pacing, emphasize task-relevant elements, or provide supportive cues when stress markers indicate that users are approaching states of cognitive or emotional overload.

### 4.4 Mind wandering

Mind wandering is defined as “a shift in the contents of thought away from an ongoing task and/or from events in the external environment to self-generated thoughts and feelings” (Smallwood and Schooler, 2015). This phenomenon can occur either intentionally or unintentionally, and is a common experience for everyone (Seli et al., 2016; Golchert et al., 2017). It is estimated that people spend between 25% and 50% of their waking hours experiencing mind wandering (Killingsworth and Gilbert, 2010; Kam et al., 2011). Typically, the self-generated thoughts are related to future planning and memories from the past with personal relevance, and are believed to have several benefits such as enhancing social skills and creativity (McMillan et al., 2013), and mitigating cognitive fatigue (Gouraud et al., 2017). However, as these thoughts can lead to decoupling from the external environment (Smallwood et al., 2008), they may have a negative influence on task performance (Mooneyham and Schooler, 2013; Gouraud et al., 2017). Mind wandering has been associated with poor performance in several tasks ranging from reading comprehension (Unsworth and McMillan, 2013) and working memory tasks (Mrazek et al., 2012) to driving tasks (Berthié et al., 2015).

To determine whether a participant is experiencing mind wandering, researchers typically employ the experience sampling approach, which refers to different ways of collecting self-reported measures. For example, prior work (Groot et al., 2021) has used the probe-caught method whereby participants are intermittently interrupted and probed to report whether the content of their thoughts is unrelated to the task they are asked to perform. However, a main limitation of experience sampling is that it can be obtrusive, and it does not allow researchers to observe the changes in the conscious state of participants in real time. Given the implications of mind wandering in task performance, recent research efforts seek to identify methods for probing the experience of mind wandering continuously and in an unobtrusive manner. As with other aspects of cognitive state, the detection of mind wandering can be done with a range of physiological measurements, including measurements of EEG, fNIRS, HR, and oculometric behavior.

#### 4.4.1 Assessing mind wandering with EEG

Several EEG studies have been exploring ways of detecting mind wandering as studying this phenomenon will allow us to better understand its relation to impaired performance in a variety of tasks (Dong et al., 2021) and its effects in education (Dhindsa et al., 2019). Typically, participants are engaged in a sustained attention to response task (SART), such as a visual or auditory oddball paradigm, while EEG signals are recorded, and they are asked at random intervals to report any episodes of mind wandering. The most common finding has been an elevation in alpha activity during periods of self-reported mind wandering (Groot et al., 2021; Rodriguez-Larios et al., 2021), which often precedes declines in task performance (O'Connell et al., 2009). Although the elevation in alpha activity is typically observed globally in the brain, (O'Connell et al. 2009) indicated that it is more prominent in the right inferior parietal cortex, a region that has been associated with top-down control of attention (Husain and Nachev, 2007). Another well-reported measure in the literature for detecting mind wandering is the decrease in the ERP amplitude P300 (Smallwood et al., 2008; O'Connell et al., 2009; Baldwin et al., 2017; Kam et al., 2012; Groot et al., 2021), which requires participants to be engaged in a secondary task (e.g., responding to auditory probes) and, thus, cannot be translated well into real-world settings, as it may have a negative impact on users' performance.

While the majority of studies have reported a strong association of mind wandering with increases in alpha oscillations, Dhindsa et al. (2019) found that the effects of mind wandering on frequency band powers could not be detected at the group level. Instead, mind wandering episodes could only be detected when a non-linear SVM had been trained on an individual basis using spatial patterns of frequency bands. Moreover, a few studies have found other ERP components apart from P300 (e.g., P100, N100, contingent negative variation) to be sensitive to mind wandering (O'Connell et al., 2009; Kam et al., 2012), although it is still unclear if these findings depend on the exact task employed for generating the ERPs. A new approach has been recently proposed for detecting the occurrence of mind wandering based on the presence of a spatiotemporal slow EEG wave, which resembles slow waves observed at transitions to sleep (Andrillon et al., 2021). However, it has been speculated that the task employed by Andrillon et al. (2021) is not engaging enough, potentially inducing drowsiness rather than purely capturing mind wandering states. Therefore, further research is required to generalize these findings to experimental contexts with more demanding tasks.

#### 4.4.2 Assessing mind wandering with fNIRS

Durantin et al. (2015) were the first to demonstrate the feasibility of detecting mind wandering with fNIRS. In this study, participants performed the SART where they were asked to look at a sequence of digits presented on the screen and press a button for every digit presented except for number 3, which served as the target stimulus. Mind wandering was defined as those periods during which participants incorrectly pressed the button for the target stimulus, given the association of mind wandering with disrupted performance during the SART (Mooneyham and Schooler, 2013). Their results showed relatively high levels of oxygenated hemoglobin in the medial prefrontal cortex during episodes of mind wandering (Durantin et al., 2015). Note that the increased activity in the medial prefrontal cortex, a primary brain area of the default mode network (DMN), is consistent with the well-established association of DMN with mind wandering revealed in fMRI studies (Christoff et al., 2009).

Furthermore, in the study by Durantin et al. (2015), fNIRS signals at periods around the infrequent (target) stimuli were used in a Linear Discriminant Analysis (LDA) and were found to predict whether the participants wrongly responded to a trial or correctly withheld from responding, with classification accuracy above chance levels (56%), obtained using a binary LDA classifier evaluated with cross-validation on trial-level fNIRS features. Although it is unclear how motor-related brain activity related to pressing the response button may have influenced the classification, the findings suggest that mind wandering may be able to be detected from fNIRS signals. Consistent with Durantin et al. (2015), a subsequent study that also utilized the SART, reported increased HbO2 levels in the bilateral prefrontal cortex during mind wandering, but also decreased HbR levels (Liu et al., 2021). In addition, it showed superior performance of LDA, when combined with a subject-based time window selection algorithm, in detecting periods with mind wandering (F1-score of 73%), with this performance obtained using a binary classifier evaluated through cross-validation on each participant's fNIRS time-windowed features.

Finally, a recent study by Ogihara et al. (2022) showed the potential of fNIRS-based functional connectivity to distinguish mind wandering from on-task episodes during real driving. Although the exact topography of functional connectivity that assisted with the classification varied significantly across individuals, some of the most prominent features across individuals were connections between regions of the dorsal and ventral attention networks (e.g., middle frontal gyrus, dorsolateral superior frontal gyrus) that have been implicated in endogenous attentional control (Doricchi et al., 2010; Japee et al., 2015).

#### 4.4.3 Assessing mind wandering with HRV

Several studies have examined how autonomic activity, as indexed by HR and HRV, changes during mind wandering. In one of the earliest investigations, Smallwood et al. (2007) showed that episodes of task-unrelated thought were accompanied by increased HR, indicating heightened physiological arousal during mind wandering. This finding aligns with evidence from Ottaviani et al. (2015), who reported that mind wandering is associated with reduced vagal influence on the heart, reflected in decreases in the HF component of HRV and, in some cases, increases in sympathetic markers such as the LF/HF ratio. These autonomic changes suggest a shift toward sympathetic dominance during episodes of off-task thought.

Beyond moment-to-moment fluctuations, HRV has also been linked to individual differences in the tendency to engage in spontaneous, self-generated thought. Nashiro et al. (2022) found that lower resting HRV was associated with a greater propensity for mind wandering, suggesting that trait-level autonomic regulation may influence the frequency or intensity of internally oriented thought. Taken together, these findings indicate that both state- and trait-level variations in HR and HRV carry useful information for detecting mind wandering, although additional work is needed to identify the most reliable HRV markers across tasks and populations.

#### 4.4.4 Assessing mind wandering with EDA

EDA has also been employed in detecting mind-wandering states in different contexts, often related to education. In this respect, Blanchard et al. (2014) conducted a study in which students were asked to read texts on scientific methodology. They combined features of the EDA signal with others related to skin temperature and context (e.g., text difficulty), and although the classification results were modest, they highlighted the informative potential of EDA for this purpose. More recently, Brishtel et al. (2020) built a Random Forest classification model that was able to detect mind wandering states of students while reading scientific texts from the EDA signal. This model yielded F1 = 78% when only features of this signal were used and 83% when combined with eye-tracking features, with these values obtained from a binary mind-wandering classifier evaluated using k-fold cross-validation across participants. On the other hand, in a meditation context, Cheetham et al. (2016) obtained an AUC of 81% in detecting mind wandering using only EDA signal features, based on a supervised classifier evaluated using cross-validation on windowed EDA segments.

#### 4.4.5 Assessing mind wandering with eye-tracking

Mind wandering involves a shift of attention from external, task-specific information to internal, unrelated thoughts or ideas (Hutt et al., 2019). Unlike other cognitive features, mind wandering can occur without conscious awareness (Smallwood and Schooler, 2015), making it challenging to measure accurately. Eye-tracking is a promising method for detecting mind wandering due to the strong connection between attention and eye movements, as evidenced by decades of research.

Although eye-tracking information is commonly used to gauge attention, substantial research has identified changes in eye movements when individuals are not focused on visual tasks, such as during mind wandering. There appears to be a shift in the visual system's sampling strategy during mind wandering, with fewer regions being sampled and incoming information being limited by blinks. This aligns with findings that show reduced cortical processing of external stimuli during mind wandering (Baird et al., 2014).

Observing eye-related behavior, self-reported mind wandering during reading is linked to fewer and longer fixations, increased variability in fixation patterns, and more frequent eye blinks (Faber et al., 2018; Uzzaman and Joordens, 2011). Similarly, mind wandering during visual scene processing is associated with fewer and longer fixations, greater dispersion of fixations, and more frequent eye blinks (Krasich et al., 2018). Therefore, mind wandering should be assessed differently from other eye-tracking metrics, as it is akin to “looking without seeing,” where the eyes may fixate on the appropriate external stimulus, but minimal processing occurs (Skaramagkas et al., 2021). Mind wandering classification has also been assessed through eye-tracking data, demonstrating detection accuracy significantly higher than chance level (Hutt et al., 2019). Overall, the literature indicates that characteristic changes in fixations, blinks, and gaze dispersion provide informative behavioral markers of mind wandering.

#### 4.4.6 InfoVis applications for mind wandering

Mind wandering has important implications for how users interact with information visualizations, as it disrupts the allocation of attention to visual displays and reduces the processing of task-relevant information. Studies have shown that mind wandering is accompanied by fewer and longer fixations, increased gaze variability, and more frequent eye blinks during reading or scene viewing tasks (Faber et al., 2018; Uzzaman and Joordens, 2011; Krasich et al., 2018). These changes indicate a decoupling between visual input and cognitive processing, whereby users continue to look at a visualization but engage minimally with its content.

In visualization contexts, such decoupling can impair users' ability to extract key relationships, follow visual encodings, or integrate information across multiple elements. Reduced sampling of the visual field and greater blink-driven interruptions limit the amount of information acquired, increasing the likelihood of missed signals, misinterpretations, or delays in task progression. Moreover, because mind wandering often occurs without conscious awareness (Smallwood and Schooler, 2015), users may not realize that their visual attention has lapsed, making passive sensing particularly valuable.

Eye-tracking–based classifiers of mind wandering have achieved detection performance above chance levels (Hutt et al., 2019), demonstrating that behavioral signatures of attentional disengagement can be identified in real time. This opens opportunities for InfoVis systems to incorporate indicators of mind wandering (such as fixation instability, reduced gaze dispersion, or blink frequency) to monitor users' engagement with a visualization. Such systems could adapt by reducing visual complexity, highlighting key information, or pausing dynamic updates when evidence of mind wandering emerges.

Overall, the characteristic visual behaviors associated with mind wandering provide actionable cues for designing visualization interfaces that can detect when users disengage from external information and support re-engagement during complex analytic tasks.

## 5 Discussion

This comprehensive review has shed light on the diverse methodologies for assessing cognitive states through the analysis of neurophysiological signals, as well as on the role of ML in this area of research. We examined several cognitive aspects known to strongly influence user performance, namely: mental fatigue, mental workload, stress, and mind wandering. Table 1 summarizes practical trade-offs across the main sensing modalities discussed in this review (e.g., cost/complexity, portability, artifact sensitivity, and typical strengths/limitations). We also discussed strategies for assessing these processes, via non-invasive brain imaging modalities (EEG, fNIRS) and peripheral physiological recordings (HRV, EDA, and eye activity), which are essential for designing neuroadaptive InfoVis systems. Despite the promising findings reported in early studies (Maat and Pantic, 2006; Hirshfield et al., 2009), research on neuroadaptive InfoVis systems remains largely unexplored. We envision that progress in cognitive state monitoring will serve as a catalyst for pioneering adaptations in InfoVis, fostering increased user engagement and more effective decision making.

**Table 1:** Practical comparison of sensing modalities commonly used for cognitive-state-aware adaptive InfoVis.

Modality	Primary signal target	Cost	Setup complexity	Ambulatory suitability	Motion artifact sensitivity	Main strengths	Main limitations
EEG	Cortical electrical activity	Med–High	High	Med	High	Fast detection of cognitive-state changes; rich spatiotemporal signatures	High susceptibility to artifacts; lower portability than simpler peripheral sensors
fNIRS	Cortical hemodynamics	Med	Med	Med–High	Low–Med	Complementary to EEG; useful in ecologically valid tasks	Slower response due to blood-flow dynamics; limited depth (cortex)
HRV (ECG/PPG- derived)	Autonomic cardiac dynamics	Low–Med	Low–Med	High	Med	Continuous and unobtrusive; easy deployment	Limited specificity; some measures have delayed response relative to task changes
EDA	Sympathetic arousal via skin conductance	Low	Low	High	Med	Low-cost, simple setup; rich tonic/phasic feature space	Low specificity; interpretation depends on context and task conditions
Eye activity	Oculomotor/visual attention behavior	Low–Med	Low	High	Med	Ecologically valid; low intrusion; highly relevant for InfoVis behavior	Many confounds by non-target factors (e.g., illumination); careful calibration required

The ratings (Low/Medium/High) are qualitative summaries intended to support design trade-off discussions.

**Figure 3:**
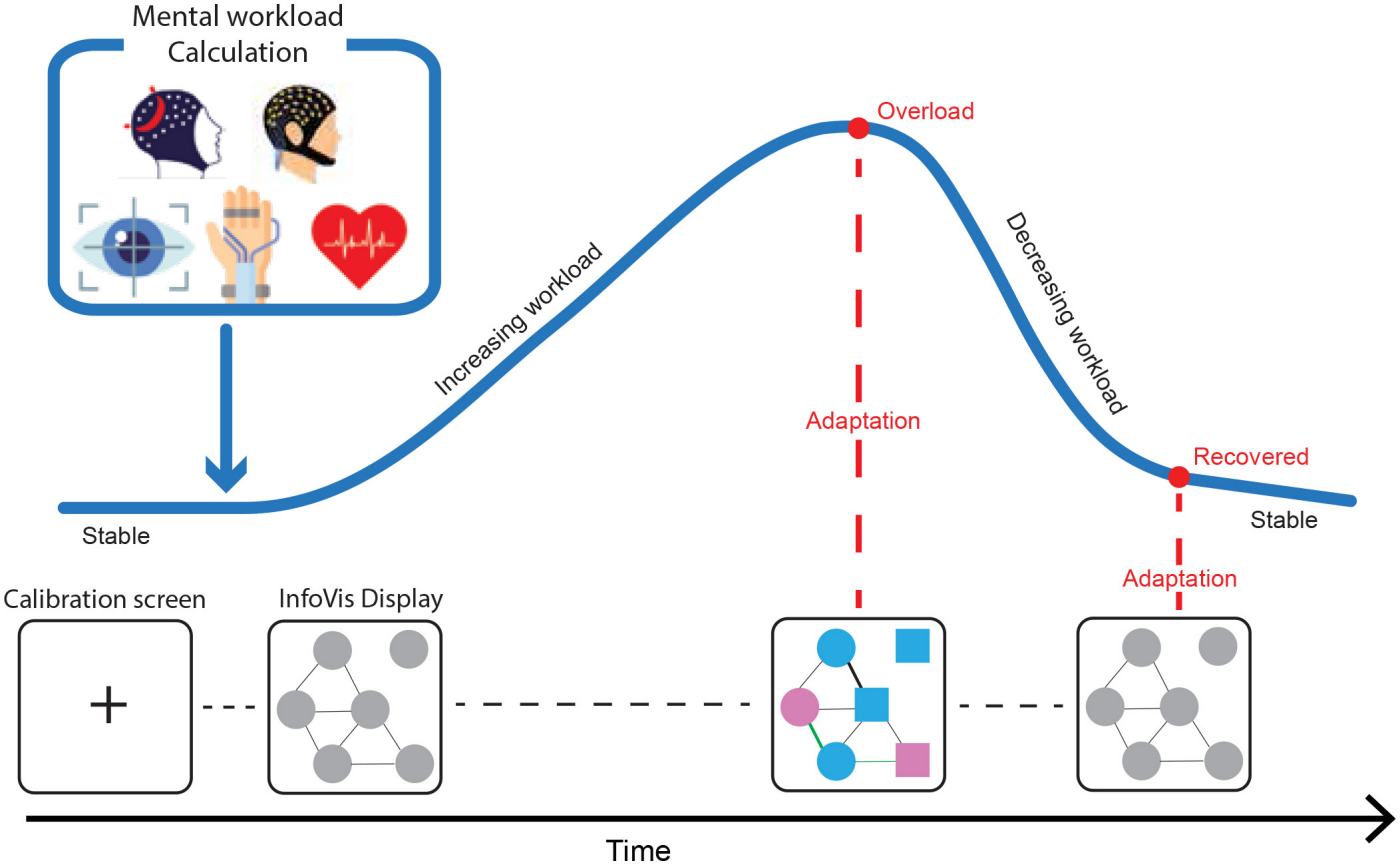
Illustrative conceptual example of workload-driven neuroadaptive InfoVis over time. A multimodal inference module estimates the user's mental workload, and the visualization is adapted when overload is detected (e.g., simplification/reduced complexity), then progressively restored after recovery/stabilization. The display snapshots are schematic and intended for explanation only. For an actual prototype that implements these concepts please see Cardona et al. (2025).

At present, there is no broadly agreed taxonomy of neuroadaptive adaptation mechanisms for InfoVis. In practice, adaptations may target different levels, including visual encoding (e.g., color encoding schemes, animation speed), layout/composition (e.g., information density, visual complexity, and layout reconfiguration), and task flow/interaction (e.g., interaction constraints, guidance, or step-wise disclosure), depending on the inferred state, task, and system goals. Figure 3 provides a conceptual example of a workload-driven neuroadaptive InfoVis system that we are developing in the European project SYMBIOTIK, where the system introduces real-time modifications to visual encodings.

### 5.1 Lessons learned

Among a range of neurophysiological techniques examined in the literature, EEG seems to be the most powerful tool for probing ongoing changes in cognitive processes, as cognitive states are shown to have different spatiotemporal signatures in EEG (Niknam et al., 2025). Moreover, due to the fact that EEG relies on the fast electrical activity of neuronal synapses for inferring brain activity, it can detect changes in cognitive state much earlier than other techniques that are based on haemodynamic signals or autonomic responses (fNIRS, HRV, etc.). A main limitation of EEG, however, is that EEG channels reflect cortical activity that arise from several regions involved in a variety of processes, and thus advanced analytical frameworks are needed for extracting useful information from this complex signal. In addition, sophisticated preprocessing strategies are needed as EEG systems are prone to motion and physiological artifacts and often the neuronal-driven component is obscured by such artifacts.

Peripheral recordings such as HRV, EDA, and eye activity also play a significant role in the cognitive-state monitoring, as they provide great surrogates of the activity in the two branches of the autonomic nervous system, namely the parasympathetic and sympathetic activity. However, as indicated by Table 2, these measures lack specificity with respect to the factor driving a given autonomic response. For example, while an increase in HR accompanied by decreases in some HRV measures (e.g., RMSSD) may indicate a shift of parasympathetic to sympathetic activity, which is a hallmark of the flight-or-fight response, we cannot be certain whether this response is caused by a stressor or elevated levels of mental workload. Therefore, for better targeted adaptations in InfoVis systems, peripherally-derived measures need to be combined with additional measures such as signals of brain activity or context-related information.

**Table 2:** Neurophysiological correlates of over- (periods with stress and high mental workload) and under- (periods with drowsiness, mental fatigue and mind wandering) arousal states.

Signal	Mental fatigue	Mental workload	Stress	Mind wandering
EEG	Decreased alpha slow-wave index: power in the alpha band divided by the power in the delta and theta bands, i.e., α/(δ+θ) (Horovitz et al., 2008; Wong et al., 2013). Decreased alpha power in occipital regions (Olbrich et al., 2009). Elevated alpha spindle rates and increased alpha spindle durations (Schmidt et al., 2009; Simon et al., 2011). Decreased P300 amplitude during auditory oddball (Samima et al., 2017).	Increased theta activity in frontal midline areas and increased alpha activity in parietal areas. Increased levels of task load index: ratio of frontal (Fz) theta to parietal (Pz) alpha power, i.e., θ/α (Holm et al., 2009). Increased levels of engagement index: ratio of beta to alpha and theta power, i.e., β/(α+θ), pooled over four electrode sites (Cz, P3, Pz, and P4). Decreased P300 amplitude in parietal areas during auditory oddball (Allison and Polich, 2008; Dehais et al., 2019; Gomarus et al., 2006; Holm et al., 2009; Kqthner et al., 2014; Miller et al., 2011).	Increased levels of frontal alpha asymmetry (increased right anterior alpha activity as compared to left anterior activity) (Lewis et al., 2007; Lopez-Duran et al., 2012; Giannakakis et al., 2015). Increased prefrontal relative gamma power: ratio of gamma (25–45 Hz) to slow rhythm activity (4–13 Hz) (Minguillon et al., 2016).	Increased alpha activity, particularly in right inferior parietal cortex (Groot et al., 2021; O'Connell et al., 2009; Rodriguez-Larios et al., 2021). Decreased P300 amplitude (Baldwin et al., 2017; Groot et al., 2021; Kam et al., 2012; O'Connell et al., 2009; Smallwood et al., 2008).
fNIRS	Increased HbO_2_ levels in left (Liu, 2014) and right (Khan and Hong, 2015; Tanveer et al., 2019a) prefrontal cortex.	Increased HbO_2_ levels (Ayaz et al., 2012; Durantin et al., 2014; Fishburn et al., 2014; Gateau et al., 2015; Harrison et al., 2014), and decreased HbR levels (Lukanov et al., 2016; Midha et al., 2021; Peck et al., 2013).	Increased HbO_2_ levels in dorsolateral prefrontal cortex, inferior frontal gyrus, superior parietal cortex and orbitofrontal cortex (Rosenbaum et al., 2018; Schaal et al., 2019). Enhanced connectivity between left and right primary motor areas (Shi et al., 2020).	Increased HbO_2_ levels (Durantin et al., 2015) and decreased HbR levels (Liu et al., 2021) in medial prefrontal cortex.
HRV	Increased low-frequency power of HRV, decreased HF power and increased LF/HF (Chua et al., 2008; Goffeng et al., 2019; Qin et al., 2021; Tran et al., 2009; Zhang and Yu, 2010). Decreased RMSSD and SDNN (Goffeng et al., 2019).	Increased heart rate and decreased SDNN, RMSSD and HF power of HRV (De Rivecourt et al., 2008; Durantin et al., 2014; Engstrm et al., 2005).	Increased heart rate and LF/HF (Clays et al., 2011; Dimitriev et al., 2008; McDuff et al., 2014; Pereira et al., 2017), and decreased SDNN, RMSSD and HF power of HRV (Acerbi et al., 2017; Pereira et al., 2017; Taelman et al., 2011; Tharion et al., 2009).	Increased HR during mind-wandering episodes (Smallwood et al., 2007). Decreased HF power of HR, reflecting reduced vagal activity (Ottaviani et al., 2015). Increased LF/HF ratio, indicating a shift toward sympathetic dominance (Ottaviani et al., 2015). Lower resting HRV associated with a greater propensity for mind wandering (Nashiro et al., 2022).
EDA	Decreased mean of the phasic component of EDA (Heaton et al., 2020). Decreased variability of the tonic component of EDA (Heaton et al., 2020).	Increased rate of skin conductance responses (Li et al., 2022). Increased amplitude of skin conductance responses (Li et al., 2022; Romine et al., 2022). Increased mean of the tonic component of EDA (Li et al., 2022; Romine et al., 2022). Increased complexity of EDA signal (Nardelli et al., 2022).	Increased median value of the tonic component of EDA (Greco et al., 2023). Larger area under the curve of the tonic component of EDA (normalized by the length of the session) (Greco et al., 2023). Higher maximum peak value of the tonic component of EDA (Greco et al., 2023). Increased variability of the phasic component of EDA (Greco et al., 2023). Higher value of PSD of the EDA signal in the frequency range of 0.045–0.25 Hz (Greco et al., 2023). Decreased complexity of EDA signal (physical stress) (Nardelli et al., 2022).	Decreased peak-to-peak change in skin conductance responses (Nardelli et al., 2022).
Eye	Longer and more frequent blinks (Benedetto et al., 2011). Lower saccade magnitude (Hu, 2022). Lower pupil diameter (Dawson et al., 2014).	Increased fixation duration (Menekse Dalveren and Cagiltay, 2018; Sheridan and Reingold, 2017). Increased number of fixations (He et al., 2012; Mallick et al., 2016) Increased pupil size (Mallick et al., 2016; Pomplun and Sunkara, 2019; Zhong et al., 2011) Increased pupil size std (Prabhakar et al., 2020)	Increased fixation duration (Liang et al., 2017) Increased number of fixations (Ehinger et al., 2009) Pupil size std (Baltaci and Gokcay, 2016)	Less and longer fixations (Faber et al., 2018) More frequent eye blinks (Uzzaman and Joordens, 2011)

The use of fNIRS for assessment of cognitive state has been gaining popularity (Eastmond et al., 2022) as it provides a means for monitoring brain activity in naturalistic environments. fNIRS is less sensitive to motion artifacts as compared to EEG and provides complementary information to traces of neuronal activity, albeit on a slower timescale due to blood-flow dynamics. The main approach for tracking changes in cognitive state has been the examination of HbO2 and HbR levels in the prefrontal cortex (Table 2), a region known for its involvement in working memory, executive function, and decision making. Brain activity in this region is usually studied with fNIRS also because this activity is captured with optodes on the forehead, an area typically less hairy than other ones and, hence, with a relatively higher signal-to-noise ratio of fNIRS measurements. However, recent advances in the hardware of fNIRS systems and progress in practices for data acquisition suggest that other areas of the cortex can also be explored (Pinti et al., 2019), which can provide a richer repertoire of measures for assessing cognitive states. In addition, as shown recently (Shi et al., 2020), functional connectivity between distinct brain regions can provide insights into cognitive processes, and thus may hold the key for more accurate neurophysiological measures.

Given the recent availability of low-cost physiological sensors, several researchers are examining the advantages of simultaneously acquiring multiple physiological signals (Al-Shargie et al., 2015; Chiarelli et al., 2017; Groot et al., 2021) to assess an individual's cognitive state. Multimodal techniques leverage on the strengths of each method, such as the high temporal resolution of EEG and spatial specificity of fNIRS, to provide a more comprehensive evaluation of a person;s cognitive state. As mentioned earlier, peripheral recordings (HRV, EDA, eye activity, etc.) can detect changes in cognitive state but cannot identify the specific underlying factors (e.g., an increase in cognitive workload). Nevertheless, the information about cognitive state changes obtained from these signals can potentially enhance the reliability and sensitivity of brain imaging techniques, underscoring the significance of multimodal techniques for assessing cognitive states.

Within the broader field of AI, ML offers frameworks that leverage diverse data sources with minimal human intervention. Among these, DL techniques have increasingly demonstrated their potential for assessing cognitive states. Traditional ML methods often include linear discriminant analysis (LDA) and SVM. It is important to note that while MLs are sometimes implemented with linear kernels, alternative kernel functions (e.g., the radial basis function) enable them to capture nonlinear relationships. Similarly, ensemble methods like Random Forests can also model complex nonlinear interactions, highlighting the versatility of these approaches (Wang et al., 2009).

To sum up, in recent years, there has been a significant shift in the approach to analyzing physiological recordings. The traditional method, employed in the majority of studies, entails long and complex preprocessing pipelines, followed by the extraction of descriptive features for feeding the classifiers. This process relies on expert knowledge of neurophysiology to derive meaningful and informative features. However, more recent approaches have embraced DL architectures, which can directly extract essential information from raw physiological signals, eliminating the need for preprocessing the data. Integrating DL techniques is a relatively novel concept that warrants further exploration. It holds promise as it can easily accommodate multimodal recordings and be trained per individual, thereby reducing the impact of subject variability (a pervasive and well-known issue).

### 5.2 Challenges and future directions

A persistent challenge in cognitive state estimation is the large degree of variability across individuals. Neurophysiological responses to mental workload, stress, fatigue, or mind wandering differ substantially between users due to anatomical differences, baseline autonomic tone, prior experience, and inter-individual cognitive strategies. As highlighted earlier in this review, models trained at the group level often fail to generalize to new participants, requiring subject-specific calibration sessions that increase system complexity and reduce usability. This variability complicates the development of universal neuroadaptive interfaces and underscores the need for individualized models or adaptive algorithms capable of learning user-specific patterns over time.

Another barrier to deploying neuroadaptive InfoVis systems lies in the practical constraints of data acquisition. Physiological studies typically require strict inclusion and exclusion criteria to control for factors such as medication use, sleep quality, neurological disorders, or cardiovascular conditions, all of which influence neural and autonomic signals. Devices also require careful calibration to ensure signal quality, often demanding time-consuming setup procedures (e.g., electrode placement, optode coupling, and baseline recordings). Furthermore, determining appropriate trial durations is non-trivial: tasks must be long enough to capture stable cognitive states but short enough to avoid confounds such as fatigue or habituation. These constraints limit large-scale data collection and highlight the need for protocols and hardware that are easier to deploy in real-world contexts.

As these sensing technologies become more feasible in consumer-facing systems, ethical risks and potential misuse must also be considered. Neural and psychophysiological signals are sensitive and may be misused without appropriate safeguards (e.g., privacy protections, informed consent, purpose limitation, and controls against biased or over-interpreted inferences), especially in commercial, workplace, and other high-stakes settings. Accordingly, neuroadaptive InfoVis systems should be developed with transparency, data minimization, secure governance, and human oversight.

Furthermore, neuroadaptive interfaces, which rely on brain activity, face the common challenge of lacking contextual information (Krol and Zander, 2017). User states of interest can be either momentary or enduring, such as moods or workload, and these states can be influenced by the task demands of the environment. For optimal adaptation, systems need to support users across various and changing contexts by synchronizing contextual data with the user's mental state. This synchronization enables the system to learn user behavior and responses in different scenarios, thereby determining the best ways and times to provide support (Brouwer et al., 2012). Nevertheless, tracking memory load continuously and unobtrusively remains a prevalent challenge (Gerjets et al., 2014). Although it is possible to classify varying mental workload levels under realistic instructional conditions, using e.g. appropriate EEG power analysis methods, it is still crucial to verify whether this classification accurately reflects differences in mental workload or is influenced by perceptual-motor confounds associated with different instructional conditions. This issue is particularly relevant when motor signals are not excluded from EEG analysis. Future studies should address this by designing tasks that isolate motor components, ensuring that mental workload and motor responses do not overlap.

Another challenge arises when attempting to offer universally applicable advice. Due to the “expertise reversal effect” (Gerjets et al., 2014), the same instructional material can result in different cognitive loads on users depending on the content complexity and their prior knowledge, making one-size-fits-all guidance impractical. This effect suggests the need to tailor environments to individual characteristics and experiences, distinguishing between novice and experienced users (Kalyuga, 2009). Moreover, real-world studies are crucial. Although laboratory experiments that aim at understanding the precise relationship between mental state and neurophysiology are valuable, it is equally important to develop applications and assess their practical benefits in real-life scenarios (Brouwer et al., 2015). Tasks should be as ecologically valid as possible to demonstrate their applicability and added value in real-world situations. Integrating DL and BCIs with neuroadaptive information visualization systems can address these limitations, employing novel approaches to enhance performance and generalizability.

We have discussed the importance of DL algorithms to handle raw EEG data directly, without significant preprocessing. Preprocessing steps, often based on subjective decisions by researchers, can lead to divergent results and interpretations, potentially misleading conclusions. Novel approaches in DL can improve performance and generalizability, which is a common issue with electrophysiological data. Moreover, DL techniques provide solutions to the generalization issue (Hossain et al., 2023) by using novel approaches such as Graph Convolutional Networks (GCNs), Transfer Learning, and Generative AI. Briefly, GCNs improve relational data capture by considering topological connections between electrodes when decoding EEG data (Zhang et al., 2019), while transfer learning reduces constraints for BCIs by leveraging knowledge from previously used data across related tasks (Torrey and Shavlik, 2010).

To address the common problem of small data samples, generative DL models, such as Variational Autoencoders (VAEs), Generative Adversarial Networks (GANs), and Diffusion Models, can generate or enhance training data. They have been used for data augmentation, creating synthetic but realistic samples to expand training datasets (Salakhutdinov, 2015). Recent studies have shown that generative models proved to produce synthetic data that replicated fine details of multi-channel EEG data (Mahey et al., 2023). These results included detailed analysis such as channel-channel covariance and alpha-band topographies. This approach opens a vast variety of applications to train and validate new models.
