# Correction: Cognitive state monitoring for neuroadaptive information visualization

**DOI:** 10.3389/fnhum.2026.1899366

**Published:** 2026-06-16

**Authors:** Frontiers Production Office

**Affiliations:** Frontiers Media SA, Lausanne, Switzerland

**Keywords:** neuroadaptive systems, mental fatigue, mental workload, stress, mind wandering

Author Miguel Barreda-Ángeles was erroneously assigned to affiliation: ^3^Joint Research Centre (JRC) of the European Commission, Ispra, Italy. This affiliation has now been added as a Present address: Miguel Barreda-Ángeles, Joint Research Centre (JRC) of the European Commission, Ispra, Italy.

The original version of this article has been updated.

